# Sustainable synthesis of magnetic *Sargassum siliquastrum* activated carbon loaded with NiS nanorods for adsorption of 2,4-D herbicide

**DOI:** 10.1007/s11356-024-31987-x

**Published:** 2024-01-20

**Authors:** Ibrahem M. A. Hasan, Fawzy H. Assaf, Ahmed R. Tawfik

**Affiliations:** Chemistry Department, Faculty of Science, South Valley University, Qena, 83523 Egypt

**Keywords:** Activated carbon, Adsorption, 2,4-D, Magnetic nanocomposite, NiS NPs, *Sargassum siliquastrum*

## Abstract

The upgrade of sustainable resource waste into a valuable and beneficial material is an urgent task. The current paper outlines the development of an economical, sustainable, and prolonged adsorbent derived from *Sargassum siliquastrum* biomass and its use for potent 2,4-dichlorophenoxyacetic acid (2,4-D) removal. A simple carbonization approach was applied to obtain the highly functionalized carbon structure, which was subsequently transformed into a novel magnetic nanoadsorbent. The magnetic nanoadsorbent was characterized using Fourier transform infrared spectrometer (FTIR), X-ray diffraction (XRD), scanning electron microscopy (SEM), energy dispersive X-ray analysis (EDX), Brunauer Emmett Teller (BET)-specific surface area, and vibrating sample magnetometer (VSM). The characterization results confirm the successful formation of a high specific surface area and a uniform distribution of Fe_3_O_4_/NiS NPs grafted activated carbon. The adsorption kinetics was more accurately described via the pseudo-second order model; nevertheless, the isothermal data showed that the Langmuir model was most suitable. The monolayer adsorption capacity for 2,4-D was 208.26 ± 15.75 mg/g at 328 K. The favourability and spontaneity of the adsorption process were demonstrated by thermodynamic studies. The adsorbent displayed exceptional selectivity for 2,4-D and high stability in multi-cycle use. Electrostatic attraction, π-π stacking, and hydrogen bonding were all believed to have an impact on the sorbent’s robust 2,4-D adsorption. Analyses of real tap and Nile River water samples showed little effect of the sample matrix on 2,4-D adsorption. This study presents an innovative approach for developing highly efficient adsorbent from natural biomass and offers an affordable way to recycle algal waste into beneficial materials.

**Graphical Abstract:**

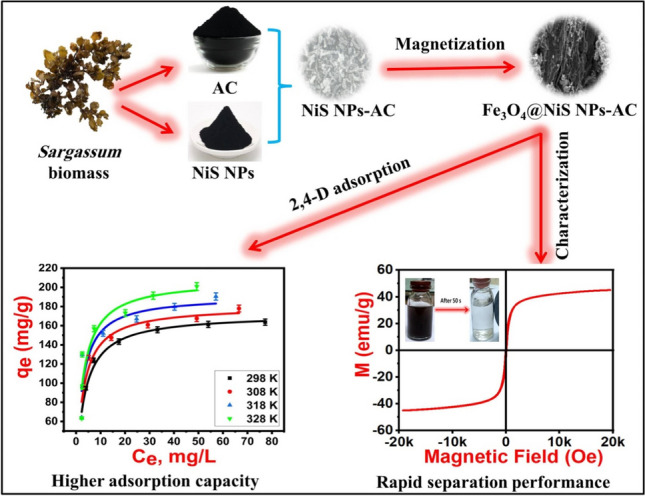

**Supplementary Information:**

The online version contains supplementary material available at 10.1007/s11356-024-31987-x.

## Introduction

One of the major global problems that have gained prominence is the availability of pure water. Agricultural, domestic, and industrial activities use more than one-third of the world’s freshwater supply, causing water pollution. Chemical pollution of natural water is a significant concern because of these human activities, which contaminate water with harmful chemicals like pesticides and fertilizers. Contaminated water supplies seriously threaten the health of people, animals, and plants unless they are expensively purified. Due to the likelihood of health risks associated with the organic and inorganic compounds they contain, surface and groundwater pollution puts humanity at threat (Salman and Hameed 2010). Water pollution is largely caused by agricultural activities, which in most regions of the world account for a significant portion of water withdrawals. Because of this, it is increasingly important to effectively remove pollutants from wastewater (Hazrin et al. 2022).

Pesticides are low-biodegradable chemical substances which influence surface and groundwater. Pesticides are usually utilized extensively for agricultural use for several goals, including inhibition of growth weeds, control of pests, plant development control, and expansion of animal and plant biomass production. To avoid their contaminating soil and water after use, they must be eliminated (Demiti et al. 2022). The low cost and high selectivity of 2,4-dichlorophenoxyacetic acid (2,4-D) make it an ideal herbicide for controlling broadleaf weeds (Georgin et al. 2021a). High water solubility, poor biodegradability, and poor soil retention characterize it. This means that it has the potential to contaminate water sources and seriously harm aquatic ecosystems. Due to 2,4-D’s economic viability and widespread consumption, there is a significant priority regarding the harm to human health brought on by exposure through inhalation, swallowing, and skin intake (Demiti et al. 2022). 2,4-D belongs to endocrine-disrupting chemicals. Seizures, digestive tract, liver, and kidney disorders, cardiac arrhythmia alterations to the endocrine and nervous systems, heat exhaustion, and low blood pressure are just a few of the adverse consequences that can occur among humans (Esteban-Arranz et al. 2018).

The highest level of 2,4-D that the World Health Organization (WHO) permits in drinking water is 20 ppm (WHO 2004). The harmful impacts generated by herbicides and their degradation products have rendered their remediation from water sources one of the most pressing ecological problems (Angın and Güneş 2021). It is seriously necessary to find a viable method to eliminate herbicides from the environment as the traditional methods of treating drinking water, such as disinfection, filtration, sedimentation, and coagulation, have shown limited effectiveness in doing so. The removal of these contaminants from water resources has involved several techniques, including advanced oxidation processes, electro-catalytic dechlorination, biological degradation, and adsorption. Adsorption procedures are used extensively in water purification owing to their low cost, efficacy, greater capacity, selectivity, regeneration, speed, and simplicity. These advantages make adsorption processes one of the most flexible and extensively employed methods for the removal of water contaminants in comparison to other techniques (Hajighasemkhan et al. 2022).

There are several types of adsorbents developed towards the removal of 2,4-D from water with good absorption capacity, e.g., 3dimensional/graphene oxide/Fe_3_O_4_ (Hajighasemkhan et al. 2022), Fe-Zr-based metal–organic frameworks (Liu et al. 2022), glutaraldehyde-crosslinked chitosan (Li et al. 2023), bifunctional porous polyethyleneimine-grafted lignin microspheres (Wu et al. 2021), modified bentonite clay (de Souza and dos Santos 2022), diethylaminoethyl cellulose (Kodali et al. 2021), and UiO-66-NMe_3_^+^ (Wu et al. 2020). Nevertheless, many of these adsorbent materials show drawbacks like, harsh preparation conditions, high energy consumption, particle agglomeration, and low adsorption capacity. Therefore, a new environmentally friendly, affordable adsorbent with a high capacity for adsorbing pollutants from water must be developed. Activated carbon (AC) represents the most widely used carbonaceous materials as adsorbents since it has high surface area, well-defined porous structure, and good adsorption capacity, as well as its low cost and ease of recovery. Thus, it can be utilized for eliminating organic contaminants from water (Demiti et al. 2022).

Conventional carbonaceous material adsorbents have difficulty removing 2,4-D because of their characteristics (Corwin and Summers 2012). The elimination of 2,4-D from water has thus been explored using a variety of modified AC, including chemically activated AC by H_3_PO_4_ (Njoku and Hameed 2011), magnetic AC (AC/Fe_2_O_3_) nanocomposite (Vinayagam et al. 2022), AC fiber modified by nitric acid (Li et al. 2018), AC-polyvinyl alcohol composite (Nawi et al. 2018), and aminosilane-grafted mesoporous carbons (Goscianska and Olejnik 2019).

In this context, scientists and researchers are focusing on hybrid AC for the elimination of 2,4-D from wastewater. NiS nanoparticles have demonstrated strong adsorption capacities towards adsorbates with favorable surface characteristics, which have been implicated in the chemisorption and physisorption of numerous dangerous pollutants (Ghaedi et al. 2014). In addition, AC has a low density which limited its ability to disperse in water. The AC, on the other hand, can offer a suitable matrix or cavity surface that regulates the growth and nucleation of NiS nanoparticles (Liu et al. 2019). Interactions of the latter with the functional groups of AC lessen nanoparticle agglomeration. Furthermore, the creation of the composite may also enhance the ability of AC to disperse in water. Carbon-based adsorbents perform exceptionally well at adsorption, but it is challenging to separate from water (Han et al. 2015). Traditional separation methods including sedimentation, coagulation, filtration, and flocculation are expensive or ineffective (Kyzas et al. 2014). An effective way to make carbon-based adsorbents capable of being effectively separated by an external magnetic field is to introduce a magnetic component such as Fe_3_O_4_ to them through a chemical co-precipitation reaction (Lou et al. 2016). Due to their distinctive characteristics such as uniform particle size, biocompatibility, and chemical stability, Fe_3_O_4_ nanoparticles are the most employed materials in the development of magnetized adsorbents (Hu and Zou 2023). It is believed that hybrid materials comprising magnetized AC cannot only become highly effective adsorbents for the removal of hazardous pollutants, but also, they will be feasible for simple magnetic separation. Various magnetic carbon-based composites have been extensively developed in recent research for the removal of different harmful contaminants from water (Hu et al. 2023; Xiong et al. 2023).

Herein, we proposed a novel, low cost, eco-friendly, and efficient adsorbent based on magnetic *Sargassum siliquastrum* AC loaded with NiS NPs for the removal of 2,4-D from aqueous solutions (Fe_3_O_4_@NiS NPs-AC). FTIR, XRD, SEM, EDX, BET-specific surface area, and VSM were employed to characterize the developed magnetic nanoadsorbent. The adsorption behavior of our adsorbent was studied in batch mode along with the impact of pH, temperature, initial concentration, adsorbent dosage, and adsorption time. With the goal of extensive information associated with the absorption mechanism of 2,4-D on the obtained magnetic nanoadsorbent, batch adsorption experiments of 2,4-D involving kinetics, isotherms, and thermodynamics studies were also performed.

## Experimental

### Gathering and algal material preparation

In May 2023, *Sargassum siliquastrum* (*SS*) alga was collected from a beach in Hurghada, Egypt, on the Red Sea (coordinates: 27°17′03″ N and 33°46′21″ E). The location was 50 to 100 m from the shore in front of the National Institute of Oceanography and Fisheries (NIOF), 5 km north to Hurghada. To prevent evaporation, seawater was added to plastic traps holding the alga as they were quickly transported to the lab. The alga was washed thoroughly with tap water to remove any external impurities and then rinsed with deionized water. After drying in the shade for 15 days, it was subsequently heated at 333 K until it attained a stable weight. After being thoroughly crushed with an electric mixer, it was then kept in storage at 277 K. 10 g of powdered alga and 100 mL of deionized water (10%) were combined in a 150-mL beaker and vigorously shaken for an hour before boiling for 15 min. The resulting *SS* extract (*SSE*) was gathered, filtered, and utilized as a capping agent to synthesize NiS NPs (Hasan et al. 2023).

### Preparation of AC

A mixture comprising 100 mL of 98% H_2_SO_4_ and 100 g of dried brown alga was slowly incorporated, and then it was permitted to stand at the ambient temperature (303 K) for 24 h before becoming stirred for 2 h. After cooling to room temperature (303 K), the reaction mixture was washed using deionized water, followed by immersion in a 2% NaHCO_3_ solution to neutralize any remaining acid. A transparent container was subsequently employed for storing AC until use after it had been dried in an oven at 373 K overnight (Esmaeili et al. 2010).

### Biosynthesis of NiS NPs

**Scheme 1:**
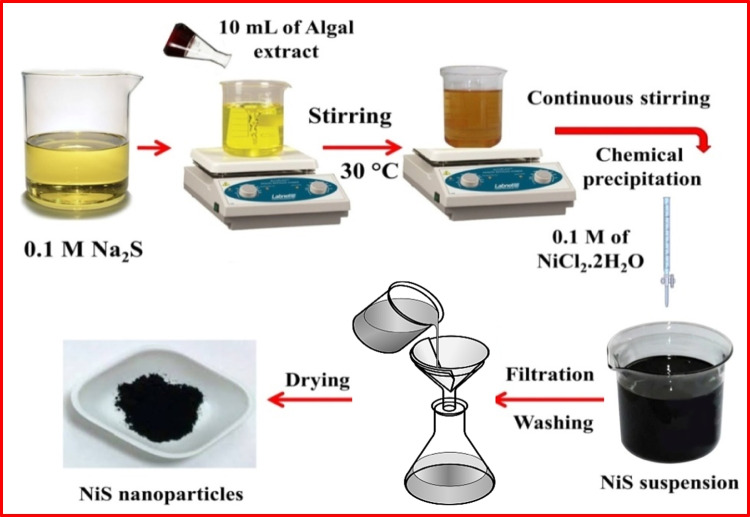
Sketch of the biosynthesis of NiS NPs using *SSE*

50 mL of 0.1 M Na_2_S and 10 mL of *SSE* were mixed at ambient temperature (303 K) and gently stirred for 2 h. The previously prepared reaction mixture was progressively incorporated with 50 mL of a 0.1 M NiCl_2_·2H_2_O solution. The magnetic stirrer was used to stir the solution for another hour to make certain the reaction was completed. The precipitate was then filtered, repeatedly rinsed with ethanol and deionized water to get rid of any remaining organic material, and dried for 4 h at 353 K (Hasan et al. 2023) (Scheme 1).

### NiS NPs-AC nanocomposite preparation

NiS NPs-AC nanocomposite was prepared according to the literature (Ghaedi et al. 2014) with a few minor adjustments. In brief, 20 mL of deionized water was used to disperse 1 g of each NiS NPs and AC, which was subsequently subjected to a 30 min ultrasonic treatment. The suspension that developed was then continuously stirred for 2 h at 353 K. The resulting black slurry was decanted and filtered to remove the liquid, and deionized water was used to repeatedly wash the solid. After obtaining the product, it was dried for 12 h at 363 K.

### Preparation of Fe_3_O_4_@NiS NPs-AC nanocomposite

**Scheme 2:**
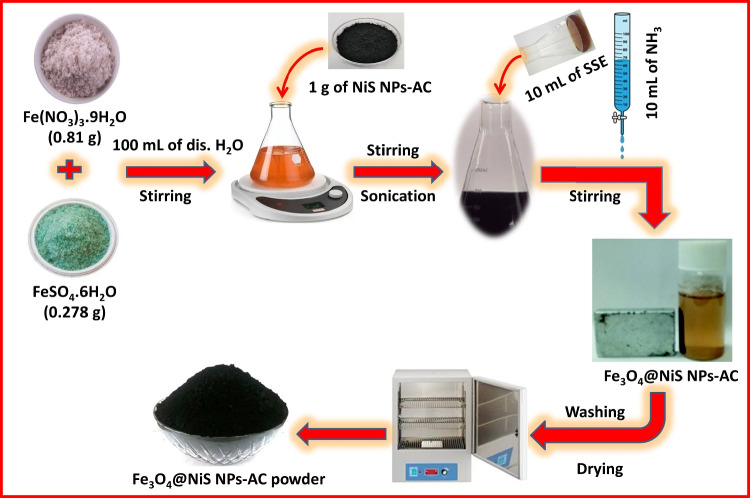
Sketch of the preparation of Fe_3_O_4_@NiS NPs-AC nanocomposite

Fe_3_O_4_@NiS NPs-AC nanocomposite was prepared by in situ precipitation of Fe_3_O_4_ NPs on the surface of NiS NPs-AC. Firstly, a mixed solution of Fe(NO_3_)_3_·9H_2_O (0.81 g, 2 mmol) and FeSO_4_·6H_2_O (0.278 g, 1 mmol) was prepared at Fe^3+^/Fe^2+^ ratio of 2:1 and stirred for 30 min. Then, 1 g of NiS NPs-AC nanocomposite was added to the pervious solution and vigorously stirred for one hour followed by ultra-sonication for 2 h at 343 K. Subsequently, 10 mL of *SSE* was added as a capping agent. The precipitation of magnetite was accomplished by the dropwise addition of 10 mL of NH_3_ solution (33%, v/v) to this solution at 353 K for 30 min. The formation of magnetite precipitate was indicated from the color change of the reaction mixture from orange to black. After that, the mixture was removed by an external permanent magnet and washed with ethanol and deionized water several times to eliminate impurities. At the end, the obtained Fe_3_O_4_@NiS NPs-AC was then dried for 6 h in an oven at 353 K. Scheme 2 provides a schematic representation of the procedure for producing Fe_3_O_4_@NiS NPs-AC nanocomposite.

### Instrumentation

The investigation focused on assessing the potential influence of phytochemicals on the development of NiS NPs and Fe_3_O_4_@NiS NPs-AC nanocomposite surface functional groups. This was accomplished by analyzing FTIR spectra within 400–4000 cm^−1^, utilizing a Shimadzu FTIR instrument, Kyoto, Japan. The XRD spectra were recorded at room temperature using Brucker D8 Advance, Germany at 2θ range of 5 to 80°. The morphology, size, and chemical composition of the nanocomposite were examined utilizing SEM (Jeol JSM-IT200, Japan) equipped with EDX from the same manufacturer. The calculation of the specific surface area was performed using the St 3 method on the NOVA touch 4LX analyzer manufactured by Quantachrome Instruments, USA. The multipoint BET method was employed to calculate the specific surface area value using N_2_ adsorption/desorption data. The average pore diameter was measured in accordance with the Barret-Joyner-Halenda (BJH) hypothesis. The magnetic properties of the prepared Fe_3_O_4_@NiS NPs-AC were examined by using VSM-1000, SES instruments PVT. LTD., India.

### Study of point of zero charge (PZC)

The pH drift method was adapted with minor modifications to ascertain PZC of Fe_3_O_4_@NiS NPs-AC (Hasan et al. 2023). A reagent bottle with a capacity of 50 mL was utilized to contain an aqueous solution of sodium chloride (0.01 M) and a fixed sorbent loading of 2.0 g/L. The pH of each bottle was subsequently modified by the addition of either a 0.1 M NaOH or HCl solution, within a pH range spanning from 1 to 11. Subsequently, the mixture was agitated for 48 h to attain equilibrium. The pH_f_ was measured to calculate the final reading for each solution. The objective of this analysis is to graphically represent the relationship between the initial pH (pH_i_) and the calculated pH difference between pH_i_ and pH_f_ (*Δ*pH). PZC is the specific location at which *Δ*pH is equal to zero. The pH meter was calibrated at pH values of 4, 7, and 10 prior to the starting of each experimental trial.

### Adsorption experiments

Adsorption experiments were conducted through a batch-type process. The one-by-one studied variables were initial pH (2, 3, 5, 7, 9, and 11), adsorbent dosage (0.01–0.125 g), contact times (up to 8 h), solution temperature (298, 308, 318, and 328 K), and initial 2,4-D concentration (50, 75, 100, 125, 150, 175, and 200 mg/L). In each set of experiments, a known amount of Fe_3_O_4_@NiS NPs-AC is mixed and shaken with 100 mL of 2,4-D with known concentration for a specific time at a specific temperature at 250 rpm using a temperature-controlled shaker. All concentrations were measured using a UV–Vis spectrophotometer (PG Instruments, model T80, UK) at 283 nm. The initial pH values of the solutions were adjusted by adding dilute NaOH or HCl solutions and were measured with a pH meter (Mettler Toledo S220, Columbus, OH). The removal percentage of 2,4-D was calculated according to the following equation:

1 $$\mathrm{Removal\; \%}=\left({C}_{o}-{C}_{e}\right)/{C}_{o})*100$$

where *C*_*o*_ and *C*_*e*_ mg/L are the initial and equilibrium concentrations of 2,4-D, respectively. For the adsorption isotherms and the investigation of the effect of initial concentration on 2,4-D removal, 0.075 g Fe_3_O_4_@NiS NPs-AC was contacted with a 100 mL 2,4-D solution with different initial concentrations (50, 75, 100, 125, 150, 175, and 200 mg/L) at pH 5. The flasks were agitated at 250 rpm and maintained at 298 K for 3 h until the equilibrium was reached. The suspensions were filtered, and 2,4-D concentrations were measured using a UV–Vis spectrophotometer. The 2,4-D uptake was calculated at equilibrium, *q*_*e*_ (mg/g), by the following equation:

2 $${q}_{e}=\left({C}_{o}-{C}_{e}\right)*\left(V/W\right)$$

where *V* (L) is the volume of the aqueous 2,4-D solution and *W* (g) is the weight of Fe_3_O_4_@NiS NPs-AC used. After collecting equilibrium data, different isotherm models (Langmuir, Freundlich, Temkin, and Dubinin-Radushkevich (D-R)) were used to fit the data. To study the kinetics of adsorption, 0.075 g of Fe_3_O_4_@NiS NPs-AC was continuously shaken with 100 mL of 2,4-D solution (100, 125, and 150 mg/L) at the optimal pH. The contact times selected fell between 1 and 8 h. Supernatant concentrations of the pesticide 2,4-D were measured at a variety of times. The results of the pseudo-first order, pseudo-second order, and Elovich models were used to calculate the adsorption kinetics from the experimental data. The rate determining step was examined using different mass transfer models such as phenomenological external mass transfer (EMT), Mathews and Weber (M&W), phenomenological internal mass transfer (IMT), and Weber and Morris (W&M) models. The thermodynamic feasibility and nature of the adsorption process were evaluated by calculating three thermodynamic parameters: the change in Gibbs free energy (*∆G°*), enthalpy (∆*H°*), and entropy (∆*S°*).

### The analysis of data and error functions

Adsorption isotherm and kinetic data were obtained through nonlinear regression analysis using OriginPro 9.0 software. The experiments were conducted in triplicate to ensure reproducibility, and the data are presented as the mean ± standard error. The error function is widely regarded as the most effective optimization technique for evaluating the degree of fit between an equation and experimental data. In addition to the regression coefficient (*R*^2^) and adjusted regression coefficient (adjR^2^), three error functions, namely the sum of square error (*SSE*), reduced chi-square test (*χ*^2^), and mean square error (*MSE*) were computed to assess the optimal alignment between the modeled equation and the empirical data (Table S6, Eq. S20-24). Furthermore, normalized standard deviation (*Δ*q%, Eq. S25, Table S6) and mean relative deviation (MRD%, Eq. S26, Table S6) were used to compare the compatibility of kinetic and mass transfer models.

### Effect of interfering ions

The impact of interfering anions (Cl^−^, NO_3_^−^, SO_4_^2−^, and PO_4_^3−^) on the adsorption of 2,4-D at 100 mg/L was studied. Different anion concentrations (0, 50, 100, and 150 mM), Fe_3_O_4_@NiS NPs-AC dosage of 0.75 g/L, temperature of 298 K, contact period of 4 h, and pH of 5 were used for these tests. Then, the amount of 2,4-D adsorbed was calculated as previously described.

### Real water applications, reusability, and stability

The adsorption capabilities of Fe_3_O_4_@NiS NPs-AC were assessed using deionized water, tap water, and Nile River water. A known quantity of 2,4-D was spiked into the samples. Real tap and Nile River water samples were gathered from Qena governorate, Egypt. The collected samples were filtered and then stored at 277 K before being employed in the adsorption experiments. Table S7 lists the physicochemical properties of both samples. The previously optimized batch adsorption procedures were utilized (100 mg/L 2,4-D concentration, Fe_3_O_4_@NiS NPs-AC dosage of 0.75 g/L, temperature of 298 K, contact period of 4 h, and pH of 5). The removal of 2,4-D was calculated as previously described.

The ability of an adsorbent to be regenerated and reused is essential in practical and large-scale applications. To evaluate the durability of Fe_3_O_4_@NiS NPs-AC, regeneration and reuse studies were conducted under the optimal conditions for the adsorption of 2,4-D during five successive cycles. After each run, Fe_3_O_4_@NiS NPs-AC nanocomposite was collected by centrifugation, cleaned three times with deionized water and ethanol, dried for two hours at 353 K, and then added to the next run.

To assess the stability of Fe_3_O_4_@NiS NPs-AC in an aqueous environment, an iron leaching test was conducted after each cycle of reusability at the optimized conditions. The solution was then subjected to centrifugation, and the supernatant was analyzed for iron content using atomic absorption spectrometer (AAS) (PerkinElmer Model 3110, USA) using air acetylene flame. Average values of three replicates were taken for each determination.

## Results and discussion

### Characterization of Fe_3_O_4_@NiS NPs-AC

#### FTIR

**Fig. 1:**
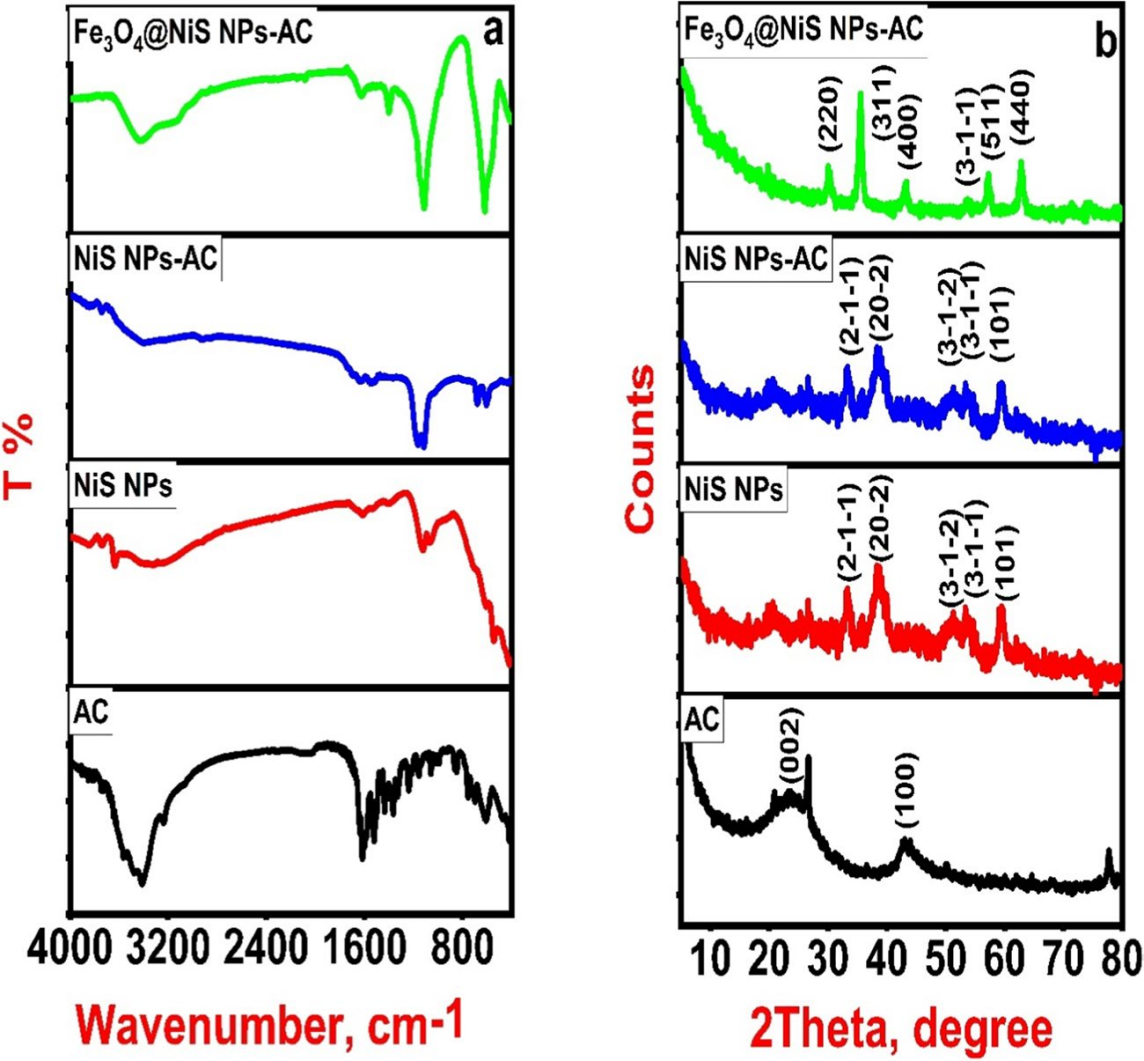
FTIR (**a**) and XRD (**b**) of the prepared AC, NiS NPs, NiS NPs-AC, and Fe_3_O_4_@NiS NPs-AC

FTIR is a powerful, adaptable, and non-destructive analytical method for chemically characterizing different compounds. It can provide valuable information about the functional groups present on the sample's surface. Herein, AC possessed numerous bands, as shown by the FTIR spectrum, demonstrating its complexity (Fig. 1a, black line). The three bands at 3550.31, 3483.78, and 3412.42 cm^−1^ were due to the NH or − OH stretching vibrations (Yamil et al. 2021). Moreover, the bands found at 3235.97 and 2925.48 cm^−1^ were assigned to the symmetrical and asymmetrical stretching vibrations of C−H bond of methyl and methylene groups (Georgin et al. 2020). Additionally, the stretching vibration of C−C = O group was responsible for the weak band at 2130 cm^−1^. The stretching vibrations at 1638.23 and 1617.02 cm^−1^ were attributed to the C = O and C = C groups, respectively (Georgin et al. 2021b). The FTIR band at 1399 cm^−1^ is due to − OH bending vibration (Lima et al. 2019). The C−O stretching vibration bands of primary and secondary alcohols were exhibited at 1160.94 and 1116.58 cm^−1^, respectively (Cunha et al. 2020; Thue et al. 2020). The vibrational band at 613.25 cm^−1^ is caused by the NH_2_ bending vibration (Lima et al. 2021). Figure 1a (red line) displays the FTIR spectrum of NiS NPs. The two broad bands at 3421 and 1631 cm^−1^ correspond to stretching and bending vibrations of hydroxyl groups on the sample's surface, respectively. Furthermore, the characteristic peaks of Ni−S appear at 1122 and 1066 cm^−1^ (asymmetric vibration). The Ni–S symmetric stretching mode can be also observed at 606 and 542 cm^−1^ (Reddy et al. 2020). After loading NiS NPs on AC, the FTIR spectrum of NiS NPs-AC (Fig. 1a, blue line) seems to be like that of NiS NPs with a little change in peak position and intensity, demonstrating that AC particles are fully covered by NiS NPs. In addition, the FTIR spectrum of Fe_3_O_4_@NiS NPs-AC appears in similar manner as NiS NPs-AC; otherwise, the peaks characteristic to NiS NPs and Fe_3_O_4_ from 1000–500 cm^−1^ are overlapped with each other and the appearance of a weak peak at 418 cm^−1^ which assigned to Fe−O bending vibration of magnetite (Fig. 1a, green line). These peaks highlight the strong binding between these nanoparticles and AC. Overall, FTIR results evaluated the successful loading of NiS NPs on AC and the subsequent deposition of magnetite nanoparticles through in situ precipitation process.

#### XRD

The crystal structure and phase formation of the synthesized samples are examined using powder X-ray diffraction. The graphitic structure of AC derived from *SS* is revealed by two characteristic peaks at 23.4° and 42.2°, respectively, indexed to the (002) and (100) crystal planes (Alswat et al. 2023) (Fig. 1b, black line). Meanwhile, the XRD pattern of NiS NPs shows five characteristic diffraction peaks at 2θ = 32.28°, 37.45°, 50.26°, 52.69°, and 59.55° which can be ascribed to the (2–1-1), (20–2), (3–1-2), (3–1-1), and (101) planes of Millerite Rhombo.R.axes β-NiS by comparing with standard data in COD 1011038 (Fig. 1b, red line) (Reddy et al. 2020). The spacing group is found to be (R 3 m (160)), and the lattice parameters are (*a* = *b* = *c* = 5.64000). Debye-Scherer’s equation was used to calculate the main crystallite size of the synthesized NiS NPs which was found to be 10.95 nm. In addition, the absence of any impurity peaks proves the high purity of the sample and adds an important advantage to the proposed method in developing NiS NPs with high purity and small crystallite size, which reflects the capping effect of *SSE*. The XRD pattern of the NiS NPs-AC nanocomposite shows no apparent AC diffraction peaks. But all the characteristic peaks agree with that of pure NiS (Fig. 1b, blue line), in clear agreement with FTIR results. The NiS NPs’ wrapping around porous carbon may be the cause of this. As a result, the carbon diffraction peaks are obscured by the strong characteristic peaks of NiS, suggesting a strong chemical bond between NiS NPs and AC (Zhu et al. 2021; Liu et al. 2023). In addition, the lack of any extra peaks resulting from impurities demonstrates the high purity of the NiS NPs-AC nanocomposite. These findings indicate that the AC substrate has no effect on the crystallinity of NiS NPs, and XRD data further show that NiS NPs have been successfully loaded on the AC surface (Zhang et al. 2016). After the in situ precipitation of magnetite nanoparticles on the surface of NiS NPs-AC, the XRD pattern of Fe_3_O_4_@NiS NPs-AC exhibited a set of new sharp diffraction peaks at 2θ of 30.06°, 35.4°, 43.2°, 57.06°, and 62.86° indexed to the 220, 311, 400, 511, and 440 planes of cubic Fe_3_O_4_ by comparison with (JCPDS card no. 19–0629) (Alswat et al. 2023). This demonstrates that Fe_3_O_4_ was successfully loaded on NiS NPs-AC. Furthermore, a weak diffraction peak at 52.69° was also appeared indicating the existence of NiS NPs (Fig. 1b, green line). All these findings imply that the in situ precipitation process successfully incorporates cubic Fe_3_O_4_ with NiS NPs-AC. In addition, the Fe_3_O_4_ structure is shown to preserve a high degree of crystallinity. The mean crystallite size of Fe_3_O_4_@NiS NPs-AC is calculated as 10.23 nm.

#### SEM

**Fig. 2:**
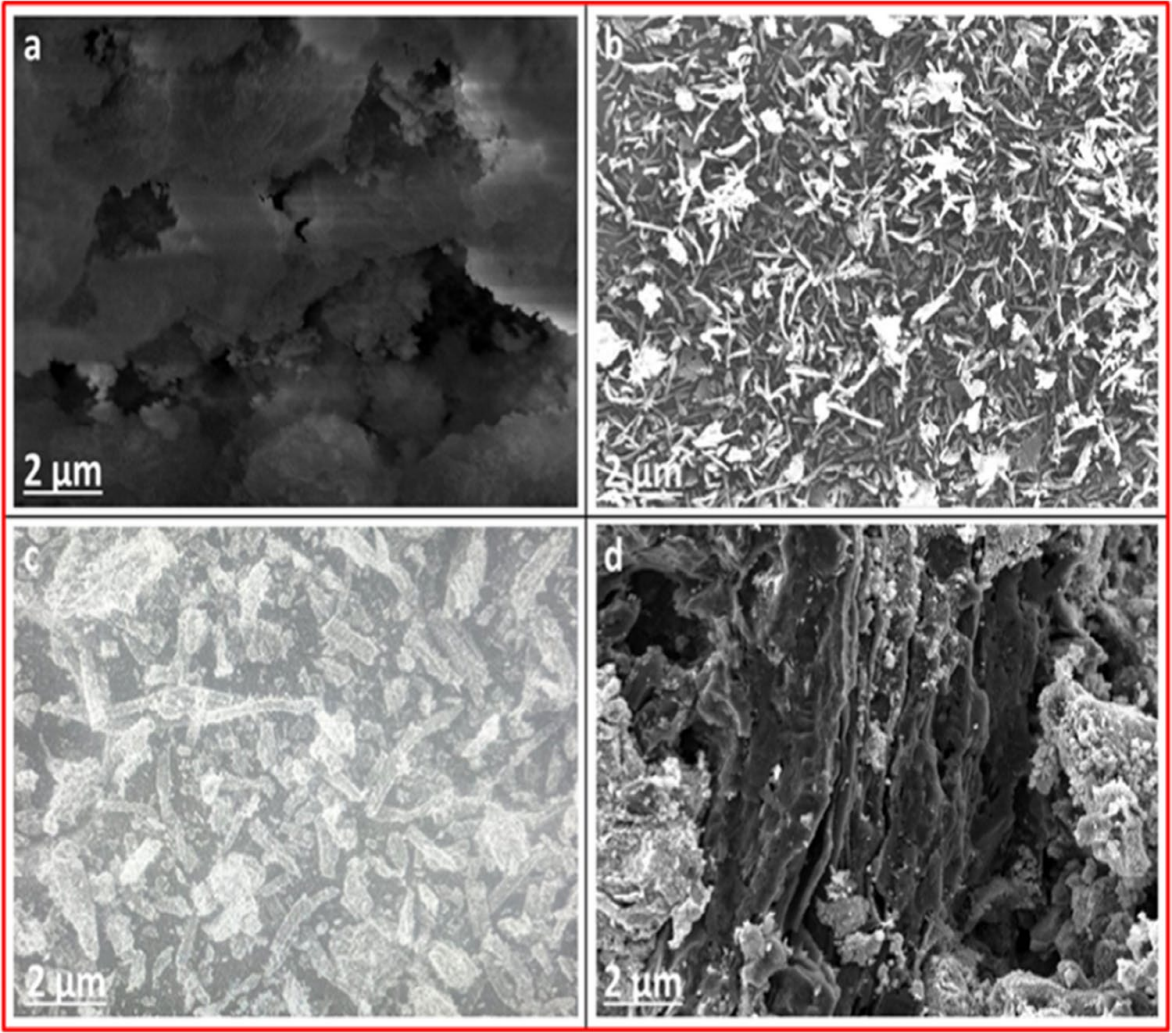
SEM images of AC (**a**), NiS NPs (**b**), NiS NPs-AC (**c**), and Fe_3_O_4_@NiS NPs-AC (**d**) at X8000 magnification

SEM characterizations were utilized to obtain more detailed information on the microstructure and morphological characteristics of the prepared samples. The results of these characterizations are presented in Fig. 2a–d. The outcomes of SEM examination of AC at X8000 magnification are depicted in Fig. 2a. An irregular, porous surface with voids was apparent in the SEM image, affording it a heterogeneous aspect. Heterogeneous surfaces incorporate higher adsorptive performance due to the abounding features and corresponding boost in surface area. Figure 2b shows the rod-like structure of NiS. The nanorods have rough surfaces, diameters in the region from 20 to 40 nm, and lengths are from 200 to 300 nm. SEM characterization provides a strong support for the consistent distribution of NiS NPs on the porous carbon structure. When comparing Fig. 2c with Fig. 2a, we recognize that the nanorods of NiS, with a diameter of about 50 nm, have entirely covered and approximately uniformly distributed on the surface of AC, making the original surface morphology of AC no longer observed, which reflects the strong binding between them (Alswat et al. 2023). On the exterior of AC, a continuous and well-defined porous NiS membrane is visible, as shown in Fig. 2c. This porous structure is well known for improving adsorption capacity (Liu et al. 2023). The surface morphology was significantly changed after subjecting NiS NPs-AC to magnetization, as shown in Fig. 2d. The Fe_3_O_4_@NiS NPs-AC nanocomposite possesses a rough and porous coral reef structure with voids and cavities. Thus, the uneven topography of the Fe_3_O_4_@NiS NPs-AC nanocomposite is expected to promote adsorption active sites for capturing more target pollutants and consequently increases adsorption rates and capacities.

#### EDX

**Fig. 3:**
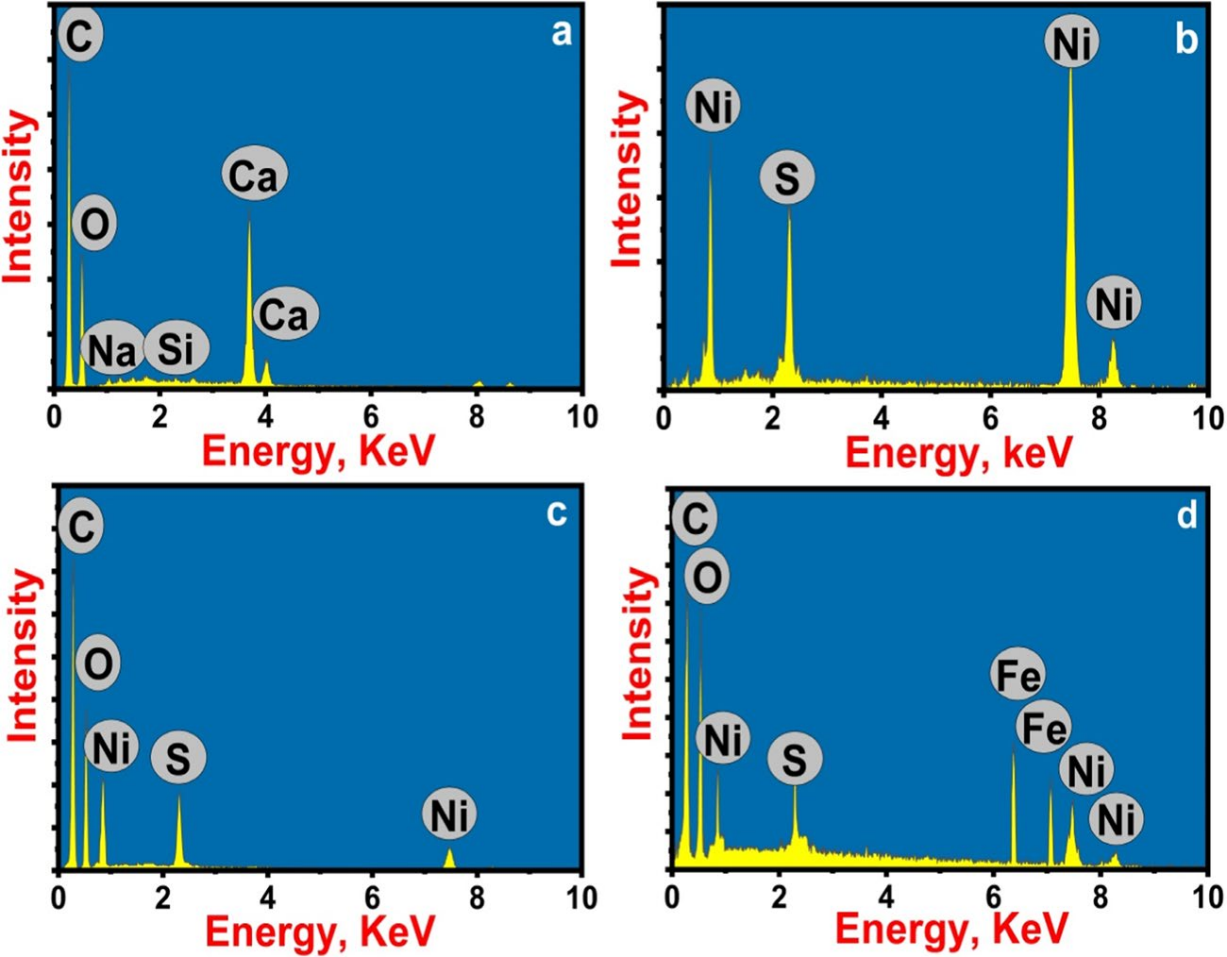
EDX profiles of AC (**a**), NiS NPs (**b**), NiS NPs-AC (**c**), and Fe_3_O_4_@NiS NPs-AC (**d**)

EDX analysis was used to determine the chemical composition of the prepared adsorbents. The EDX spectrum of pure AC confirmed the presence of C (60.32%), O (34.32%), Ca (4.76%), Si (0.1%), and Na (0.51%) by weight (Fig. 3a). As C is the element with the highest abundance, the dried *SS* seaweed sample was probably extensively carbonized. The oxygen availability demonstrates that oxygen containing species are present on the carbon surface. The distribution of C and O supports the validity of AC synthesis. The AC sample also contains trace levels of mineral elements like Ca and Na. Meanwhile, the presence of only Ni (64.08%) and S (35.92%) and the absence of other chemical contaminants are depicted in EDX spectrum of NiS NPs (Fig. 3b), indicating the high purity of the sample. Additionally, the sample had Ni:S molar ratios of 1:1. The result is in a good agreement with the chemical formula NiS and XRD results. The obtained NiS NPs-AC appeared to consist of carbon, oxygen, nickel, and sulfur in accordance with EDX spectrum in Fig. 3c, demonstrating that NiS NPs was crafted well on AC surface. Moreover, EDX results of Fe_3_O_4_@NiS NPs-AC show the presence of C, O, Ni, S, and Fe, proving the successful in situ precipitation of Fe_3_O_4_ on the NiS NPs-AC surface and the formation of Fe_3_O_4_@NiS NPs-AC nanocomposite. The weight percentages of the chemical elements in the NiS NPs-AC sample are 37.72, 24.28, 25.45, and 12.55 for C, O, Ni, and S, respectively. Comparatively, the weight percentages of C, O, Fe, S, and Ni in Fe_3_O_4_@NiS NPs-AC nanocomposite were 27.52, 34.48, 20.22, 5.35, and 12.43, respectively (Fig. 3d). The distribution of C, O, Fe, S, and Ni proved the loading of NiS–Fe_3_O_4_ NPs onto the surface of AC. Additionally, according to the EDX data, the C and O elements are the most abundant elements in the sample. This suggests that when the NiS–Fe_3_O_4_ NPs are loaded, the oxygen atoms on the AC surface form bonds with the Ni and Fe atoms. These findings concur with those from XRD and FTIR (Alswat et al. 2023).

### Specific surface area

**Fig. 4:**
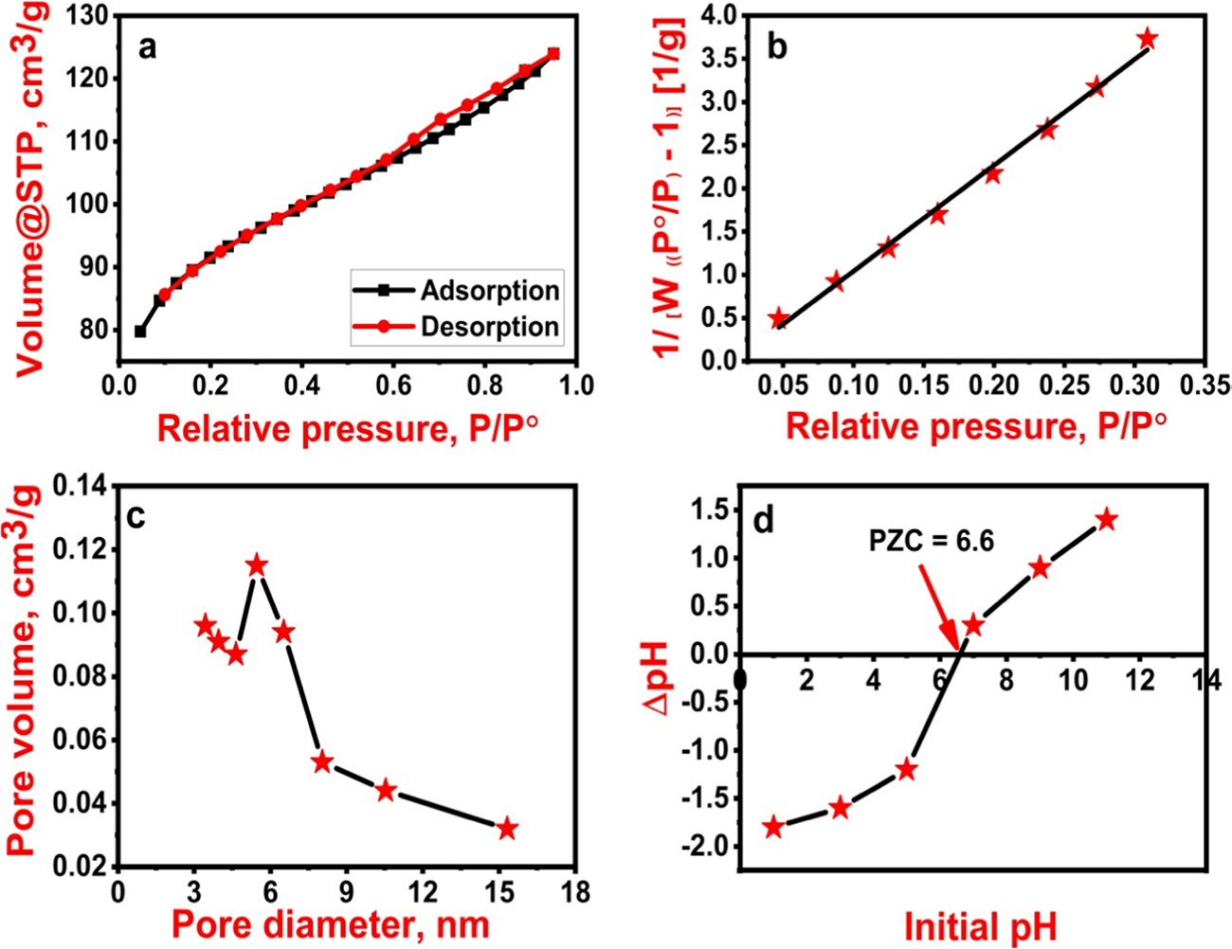
N_2_ adsorption/desorption isotherm (**a**), BET plot (**b**), BJH plot (**c**), and PZC of the prepared Fe_3_O_4_@NiS NPs-AC (**d**)

In the adsorption process, the surface area is extremely important since it provides the adsorbent with more active sites. As a result, N_2_ adsorption–desorption isotherm and BJH pore size distribution were used to examine the specific surface area and porosity (Fig. 4a–c). In the relative pressure ranges of 0.4**–**1.0 P/Po, Fe_3_O_4_@NiS NPs-AC exhibits type-IV sorption isotherm with an H3 hysteresis loop, demonstrating the existence of mesoporous structure (Fig. 4a). According to the BET results, the specific surface area is 288.439 m^2^/g with a pore volume of 0.192 cm^3^/g (Fig. 4b). The pore diameter depicted in Fig. 4c is mostly above 3.41 nm, as determined by BJH measurement. A mesoporous structure with a larger specific surface area can result in the formation of more adsorption sites. As a result, they are likely to contribute to the diffusion of adsorbates and the improvement of adsorption performance (Zhu et al. 2021).

#### PZC

The pH value whereby the surface negative charges on Fe_3_O_4_@NiS NPs-AC would be equal to its positive surface charges, resulting in a zero charge, is known as the PZC. Determining the pH ranges within which Fe_3_O_4_@NiS NPs-AC is bound to a specific kind of adsorbate would be considerably more facile with the use of PZC measurement. The PZC of Fe_3_O_4_@NiS NPs-AC is 6.6, according to the study’s findings, which are shown in Fig. 4d. This suggested that for a pH value lower than 6.6, the Fe_3_O_4_@NiS NPs-AC surface would be positively charged and have the capacity to bind negatively charged adsorbate molecules. As opposed, a working pH higher than 6.6 would promote the adsorption of positive adsorbate molecules onto the negative surface of Fe_3_O_4_@NiS NPs-AC. As a result, the electrostatic forces between adsorbate and adsorbent change significantly depending on the pH of the solution.

### VSM analysis

**Fig. 5:**
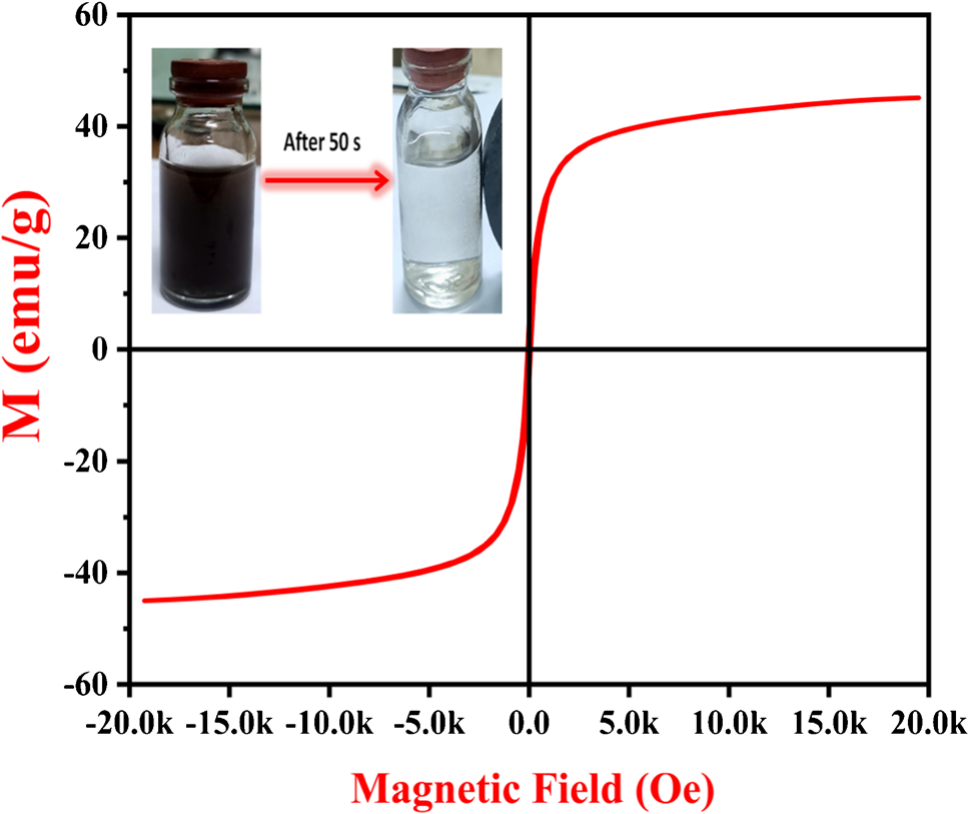
Magnetic hysteresis curve of Fe_3_O_4_@NiS NPs-AC

The adsorbent’s recovery is assisted by its magnetic properties due to the rapid separation induced by an external magnetic field (Demiti et al. 2022). The magnetic characteristics of the synthesized Fe_3_O_4_@NiS NPs-AC were determined using the VSM method by measuring its magnetization behavior as a function of the applied magnetic field between ± 20 kOe at ambient temperature. Figure 5 exhibits an S-shaped curve that passes through the origin with the adsorbent’s saturation magnetization of 45 emu/g. Likewise, the absence of hysteresis loops in the magnetization curve implies that the adsorbent has superparamagnetic nature (Hu et al. 2023; Xiong et al. 2023). This value is much greater than the previously reported magnetic adsorbents for 2, 4-D remediation; 11 emu/g (Demiti et al. 2022), 3.98 emu/g (Sayğılı and Sayğılı 2022), 2.57 emu/g (Vinayagam et al. 2022), 6 emu/g (Herrera-García et al. 2019), and 37.28 emu/g (Nethaji and Sivasamy 2017). This could be a consequence of the small size of the synthesized Fe_3_O_4_@NiS NPs-AC, as highlighted in the XRD analysis. The higher magnetization value of Fe_3_O_4_@NiS NPs-AC facilitates its quick recovery and separation from aqueous solution under the influence of a magnetic field after 50 s, as illustrated in Fig. 5. Therefore, Fe_3_O_4_@NiS NPs-AC can be used as a magnetically separable adsorbent to clean up wastewater.

### Comparison of adsorption capacity

**Fig. 6:**
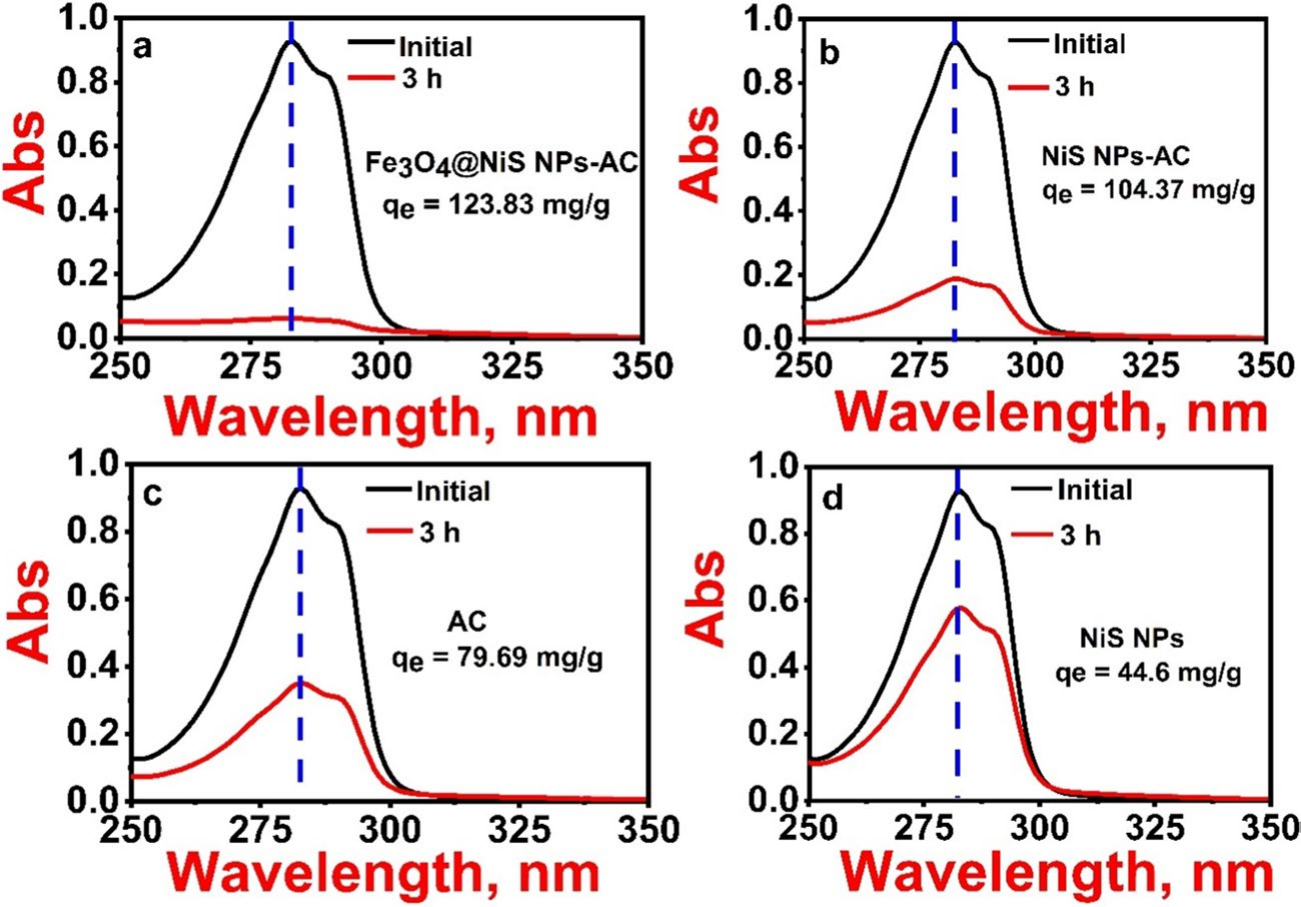
UV–Vis spectra before and after adsorption of 2,4-D with Fe_3_O_4_@NiS NPs-AC (**a**), NiS NPs-AC (**b**), pure AC (**c**), and pure NiS NPs (**d**) adsorbents (the initial 2,4-D concentration is 100 mg/L)

For comparison, the adsorption capacity of the prepared NiS NPs, AC, NiS NPs-AC, and Fe_3_O_4_@NiS NPs-AC towards 2,4-D was evaluated in this investigation (Fig. 6a–d). To perform adsorption experiments, 100 mL of a 100 mg/L aqueous solution of 2,4-D was shaken continuously for 3 h with 0.075 g of each adsorbent at 298 K. In contrast to NiS NPs, AC, and NiS NPs-AC, the 2,4-D adsorption by Fe_3_O_4_@NiS NPs-AC developed a greater decline in 2,4-D absorbance intensity at 283 nm. Interestingly, applying the Fe_3_O_4_@NiS NPs-AC delivers an excellent increase in the adsorption capacity of 2,4-D. The synergetic adsorption effect in the composite promotes this improvement. In other words, diffusion and filling through mesoporous inter-channels were stimulated by the substantial osmotic pressure that was produced by various concentration gradients of 2,4-D in the solution, in line with the earlier discussions in Figs. 2d and 4a (Liu et al. 2019; Chowdhury et al. 2021). Consequently, Fe_3_O_4_@NiS NPs-AC was used to perform further adsorption isotherm, kinetics, and thermodynamics research.

### pH study

**Fig. 7:**
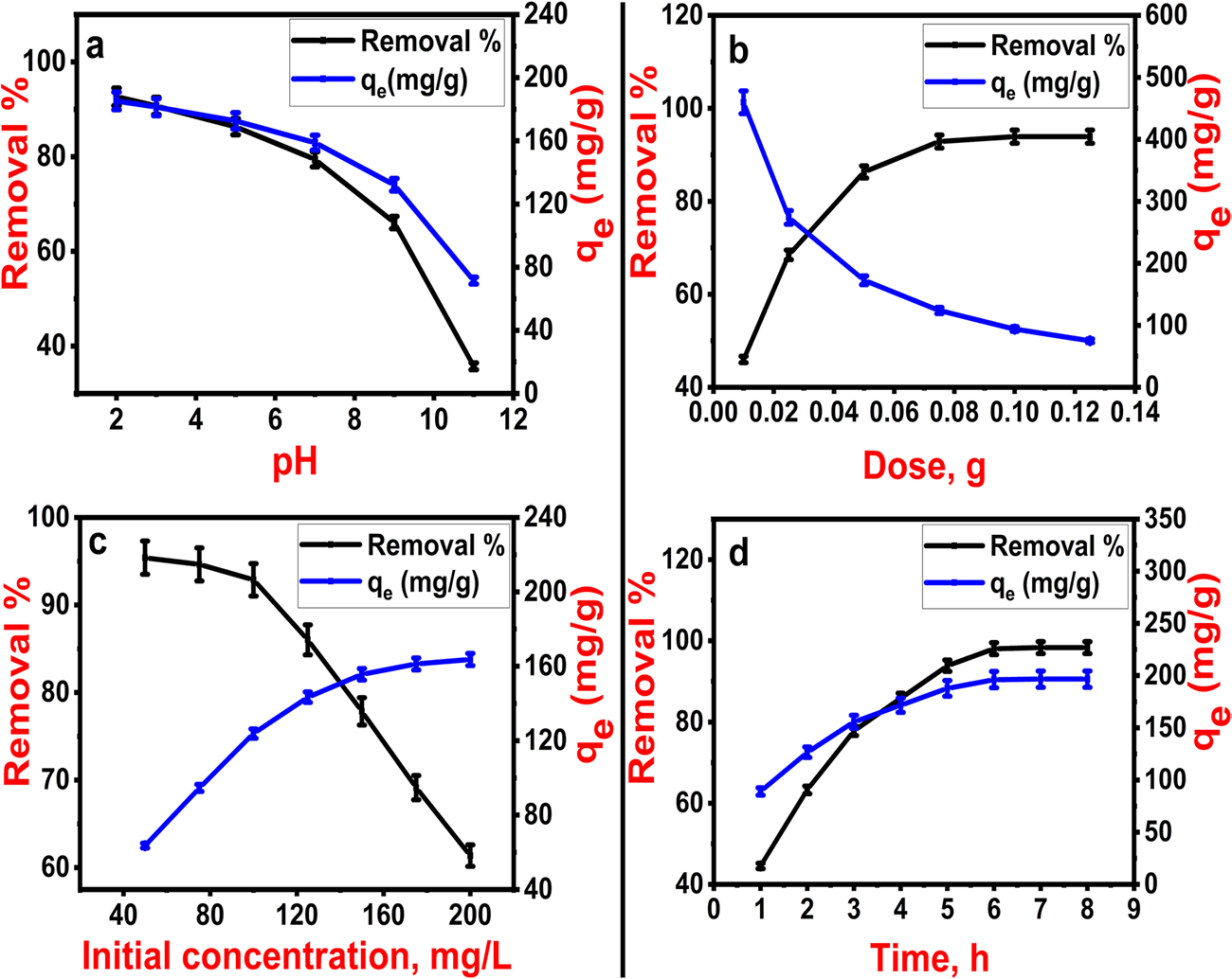
Effects of operational parameters on 2,4-D adsorption removal and capacity; pH (**a**), Fe_3_O_4_@NiS NPs-AC dose (**b**), initial 2,4-D concentration (**c**), and contact time (**d**)

Solution pH affects the features of both adsorbate and adsorbent. The electrostatic relationship that occurs between the adsorbate molecules and the surface of the adsorbent is believed to be the root cause of pH's effect (Njoku and Hameed 2011). To find the pH value that offers the maximum 2,4-D adsorption removal, the impact of pH on the adsorption efficiency of Fe_3_O_4_@NiS NPs-AC was studied. At pH 2, the maximum removal efficacy was attained (Fig. 7a). As pH rises, there is a substantial decrease in the 2,4-D removal efficiency, demonstrating that the value of the solution pH is an aspect to be considered during the adsorption process. At contact times of 3 h and pH values of 2, 3, 5, 7, 9, and 11, Fe_3_O_4_@NiS NPs-AC provided removal efficiencies of 92.45% ± 0.83, 90.37% ± 0.91, 85.51 ± 0.87, 79.53 ± 0.89, 66.29 ± 0.75, and 35.69% ± 0.77, respectively. So it follows that the removal performance drops as pH rises. The adsorbent’s characteristics, along with a PZC of 6.6, can be utilized to justify this observation. Therefore, Fe_3_O_4_@NiS NPs-AC would possess a positively charged surface at pH lower than 6.6. On the other hand, Fe_3_O_4_@NiS NPs-AC might have a surface with a negative charge above pH 6.6. In contrast, 2,4-D has a pK_a_ of 2.73 (Salomón et al. 2021). At acidic pH, 84.30% of 2,4-D is recognized in neutral form, and the remaining portion has been deprotonated according to the speciation graph created by (Yamil et al. 2021). Consequently, most of the 2,4-D becomes protonated at pH 2, where Fe_3_O_4_@NiS NPs-AC has a positively charged surface. Considering this, the highest adsorption uptake of 2,4-D at pH 2 may not only involve electrostatic attraction but also hydrogen and π-π bonds (Rambabu et al. 2021). Functional groups including C = O, C-O, and C-H have been identified, as reported in the FTIR study, and these functional groups are evidence that hydrogen bonds may be formed. But, above pH 2.73, 2,4-D would be ionized into the dichlorophenoxyacetate anion. Therefore, Fe_3_O_4_@NiS NPs-AC would have a negatively charged surface at pH levels above 6.6. This results in the lower adsorption of 2,4-D at higher pH values due to the electrostatic repulsion between 2,4-D anions and Fe_3_O_4_@NiS NPs-AC which is like observations obtained from previous studies (Njoku and Hameed 2011; Salomón et al. 2021). This clarifies the reason why the Fe_3_O_4_@NiS NPs-AC in the present investigation had the lowest removal efficiency at pH 11. The 2,4-D adsorption isotherms and kinetics are examined in the following sections of this study at pH 5.

### Influence of Fe_3_O_4_@NiS NPs-AC dosage

Figure 7b displays the impact of the amount of Fe_3_O_4_@NiS NPs-AC on the 2,4-D removal proportion. As the amount of adsorbent increased from 0.01 to 0.125 g/100 mL, the 2,4-D pesticide removal increased from 45.77 ± 0.8 to 93.77 ± 0.74%. The use of more Fe_3_O_4_@NiS NPs-AC leads to a higher 2,4-D removal percentage due to the increased surface area and available adsorption sites (Behloul et al. 2017). As opposed to the removal percentage, when the amount of adsorbent was raised from 0.01 to 0.125 g, the adsorption capacity was reduced from 460.19 ± 2 to 75.3 ± 1.5 mg/g. This is because as adsorbent dosage increased, high-energy adsorbent active sites were accessible, and a higher percentage of lower-energy active sites became occupied, which lowered adsorption capacity (Salomón et al. 2021). Figure 7b demonstrates that when Fe_3_O_4_@NiS NPs-AC is used in dosages greater than 0.075 g, the removal efficiency just slightly increases. This showed that 0.075 g was ideal for the tested circumstances, yielding a removal efficiency of 92.76 ± 0.85%. The adsorbent surface will become unsaturated if its dosage is increased, despite a slight change in removal percentage of 2,4-D. The agglomeration of Fe_3_O_4_@NiS NPs-AC particles in this situation will lead to a reduction in the total surface area (Supong et al. 2019). The dosage of 0.075 g/100 mL was therefore employed in the subsequent experiments.

### Influence of 2,4-D initial concentration

In the concentration range of 50–200 mg/L, the impact of initial concentration on 2,4-D adsorption was examined. The results of the study demonstrated that the adsorption capacity increased with a higher initial 2,4-D concentration, with an increase from 63.71 ± 1.15 to 163.68 ± 2.8 mg/g for initial 2,4-D concentrations of 50 to 200 mg/L, respectively. At higher 2,4-D concentrations, all active sites may have been used, there is a greater mass transfer driving force, and there are more interactions between 2,4-D molecules and Fe_3_O_4_@NiS NPs-AC which may have contributed to the increase in the adsorption capacity. Additionally, it was noted that an increase in 2,4-D initial concentration led to a decrease in the removal percentage (Fig. 7c). The removal percentage dropped from 95.52 ± 0.72 to 61.38 ± 1.2% as the initial concentration of 2,4-D increased from 50 to 200 mg/L. As the concentration of 2,4-D increased, the active sites on Fe_3_O_4_@NiS NPs-AC became saturated, which may be the cause of this decrease.

### Influence of batch time

Studies have been conducted to determine how contact time influences the adsorption of 2,4-D by Fe_3_O_4_@NiS NPs-AC by measuring the remaining 2,4-D concentration at regular intervals. The percentage of 2,4-D removed by Fe_3_O_4_@NiS NPs-AC at 298 K is depicted in Fig. 7d as a function of contact time. The adsorption rate rises with contact time and becomes fast in the beginning, then slows down until a state of equilibrium is attained after 6 h and retained up to 8 h. The percentage of 2,4-D removal then barely changed as contact time was further increased. Because there was more Fe_3_O_4_@NiS NPs-AC surface area accessible to 2,4-D initially, the rate has been faster. After the capacity diminishes, its removal rate is then merely governed by the speed at which 2,4-D molecules move from the exterior to the interior sites of the Fe_3_O_4_@NiS NPs-AC. The competition between the 2,4-D molecules and the decreasing number of Fe_3_O_4_@NiS NPs-AC active sites may also be responsible for the rate’s gradual decline over time.

### Effect of temperature on 2,4-D adsorption

**Fig. 8:**
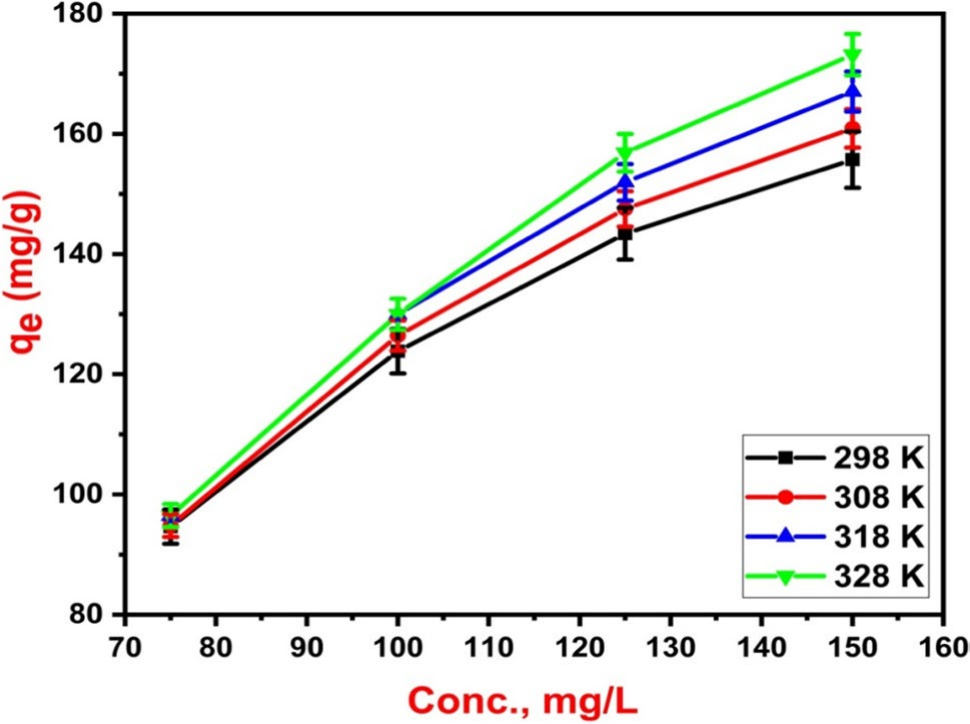
Effect of operating temperature on 2,4-D adsorption removal and capacity

Generally, higher temperatures resulted in higher removal rates and adsorption capacities across the entire concentration range (Fig. 8). This could be explained by the acquirement of the 2,4-D molecules a sufficient energy to bind to the Fe_3_O_4_@NiS NPs-AC surface at higher temperature. Furthermore, there is a rise in 2,4-D-adsorbent surface collisions that considered an indication of an endothermic adsorption process (Supong et al. 2019). In addition, a high temperature encourages a decrease in solution viscosity and an acceleration of 2,4-D molecules movement (Salomón et al. 2021). This increases the amount of 2,4-D that can be adsorbed at high temperatures by increasing the contact between the adsorbate molecules and the Fe_3_O_4_@NiS NPs-AC adsorbent (Fig. 8). However, the percentage of adsorption did not substantially rise as the temperature did. Thus, the ideal starting temperature for research may be 298 K. The adsorbent can therefore be applied to the treatment of surface water at different temperatures without losing any of its adsorbent effectiveness.

### Isotherm study

Establishing adsorption mechanisms involves the analysis of equilibrium data, which are also referred to as adsorption isotherms. Once the adsorption process attains equilibrium, such isotherms suggest how molecules are distributed across the solid and liquid phases. So, to understand adsorption phenomena, the correlation of equilibria measurements employing a mathematical or theoretical formula is imperative (Hasan et al. 2023). Using Fe_3_O_4_@NiS NPs-AC, 2,4-D pesticide adsorption examinations were conducted, and the results were analyzed using the isotherm models developed by Langmuir (Eq. S1), Freundlich (Eq. S3), Temkin (Eq. S4), and D-R (Eq. S6), respectively. Additional details regarding the nonlinear forms of these isotherm models, along with their parameters, are provided in Table S1 in the supplementary materials.

**Fig. 9:**
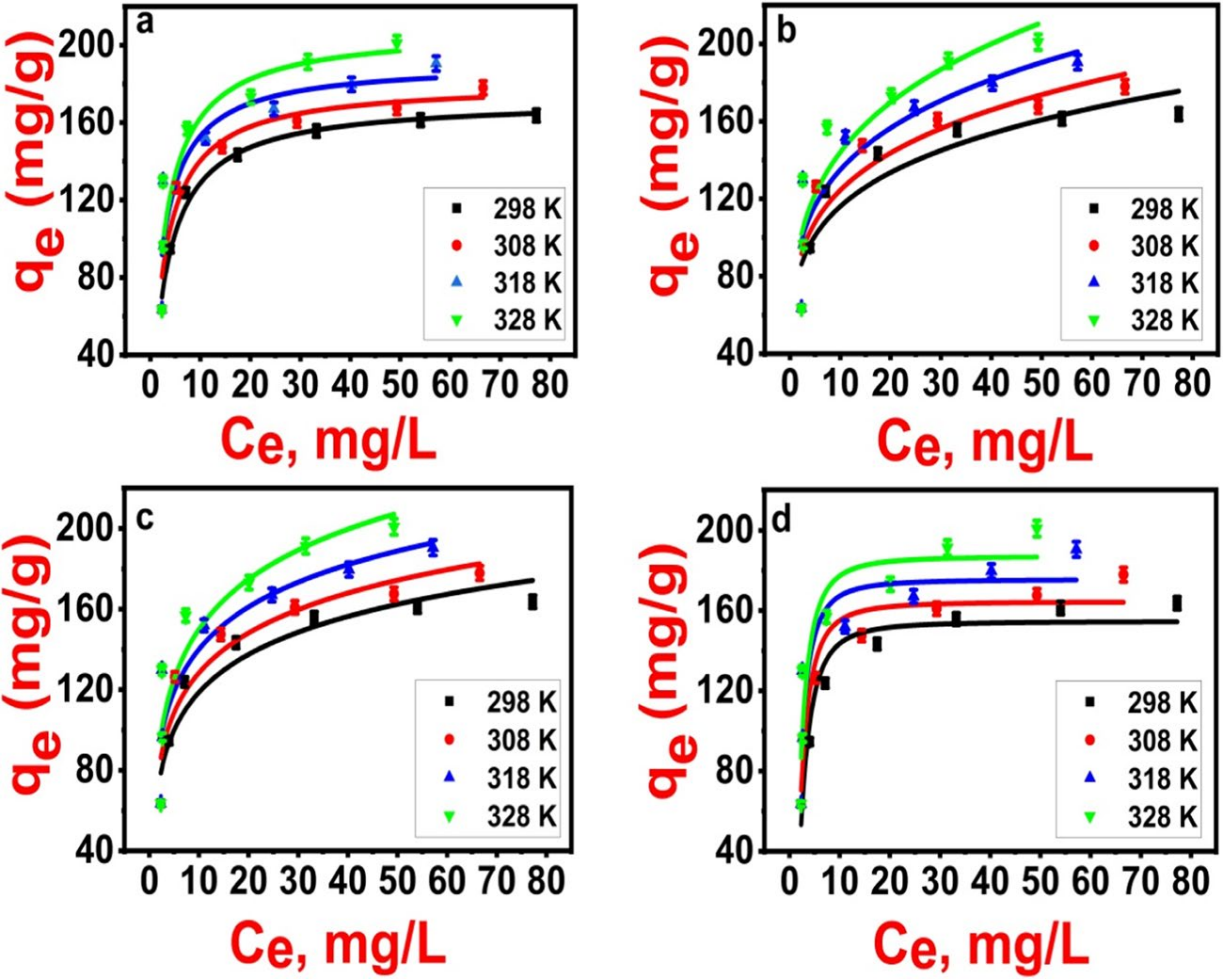
Langmuir (**a**), Freundlich (**b**), Temkin (**c**), and D-R (**d**) nonlinear plots of 2,4-D removal by Fe_3_O_4_@NiS NPs-AC at temperatures 298, 308, 318, and 328 K

Figure 9a–d displays the nonlinear graphs of various isotherm models for the adsorption of different 2,4-D concentrations onto Fe_3_O_4_@NiS NPs-AC at different operation temperatures. In addition, the different parameters relevant to each model were calculated and are listed in Table 1. Langmuir isotherm parameters *q*_*m*_ and *K*_*L*_ were 171.92 ± 2.98, 180.49 ± 7.14, 191.07 ± 14.06, and 208.26 ± 15.75 mg/g and 0.298 ± 0.024, 0.349 ± 0.062, 0.402 ± 0.127, and 0.353 ± 0.104 L/mg at 298, 308, 318, and 328 K, respectively. The high values of *K*_*L*_ indicate the high 2,4-D adsorption affinity for Fe_3_O_4_@NiS NPs-AC at the investigated temperatures. The increment of monolayer adsorption capacity with temperature revealed the favorable adsorption of 2,4-D by Fe_3_O_4_@NiS NPs-AC. The designed Fe_3_O_4_@NiS NPs-AC is beneficial for 2,4-D adsorption, as demonstrated by the values of the *R*_*L*_ (Eq. S2) which were found to be in the range of 0.013–0.063 (0 < *R*_*L*_ < 1).

**Table 1:** Adsorption isotherm constants of 2,4-D onto 0.075 g of Fe_3_O_4_@NiS NPs-AC at 50–200 mg/L of initial 2,4-D concentration, pH = 5, 3h contact time, and different temperatures (298–328 K)

Model	Parameter	Temperature (K)			
		298	308	318	328
Langmuir	*q*_*m*_ (mg/g)	171.92 ± 2.98	180.49 ± 7.14	191.07 ± 14.06	208.26 ± 15.75
	*K*_*L*_ (L/mg)	0.298 ± 0.024	0.349 ± 0.062	0.402 ± 0.127	0.353 ± 0.104
	*R*_*L*_	0.017–0.063	0.014–0.053	0.013–0.049	0.014–0.054
	*R*^2^	0.989	0.948	0.841	0.867
	*SSE*	91.1	537.39	2051.34	2042.47
	*χ*^2^	18.22	107.48	408.49	410.27
	*MSE*	4.97	35.88	104.04	104.04
	adjR^2^	0.987	0.938	0.809	0.840
Freundlich	1/*n*	0.202 ± 0.04	0.207 ± 0.06	0.214 ± 0.08	0.237 ± 0.04
	*K*_*F*_ (mg/g)	72.88 ± 9.77	77.23 ± 10.33	82.55 ± 12.83	83.49 ± 13.58
	*R*^2^	0.868	0.865	0.817	0.817
	*SSE*	1137.79	1396.98	2354.14	2825.51
	*χ*^2^	227.56	279.4	470.83	565.10
	*MSE*	55.06	80.64	126.34	137.43
	adjR^2^	0.842	0.839	0.780	0.780
Temkin	*β*	27.20 ± 3.52	28.61 ± 4.14	30.36 ± 6.03	35.89 ± 6.87
	*A* (L/mg)	7.85 ± 1.03	8.87 ± 1.26	10.08 ± 1.61	6.52 ± 0.95
	*b* (J/mol)	91.08	94.14	97.20	100.26
	*R*^2^	0.922	0.905	0.835	0.845
	*SSE*	667.43	983.54	2116.76	2386.91
	*χ*^2^	133.49	196.69	423.35	477.38
	*MSE*	27.46	55.65	116.86	109.62
	adjR^2^	0.907	0.886	0.802	0.814
D-R	*q*_*s*_ (mg/g)	154.59 ± 4.74	164.44 ± 5.45	175.52 ± 10.99	187.09 ± 11.16
	*K*	1.33 × 10^−6^	1.06 × 10^−6^	8.65 × 10^−7^	9.63 × 10^−7^
	*E* (kJ/mol)	0.61	0.67	0.76	0.72
	*R*^2^	0.944	0.941	0.824	0.859
	*SSE*	488.52	611.82	2259.71	2169.57
	*χ*^2^	97.70	122.36	451.94	433.91
	*MSE*	18.92	21.62	87.24	82.08
	adjR^2^	0.932	0.929	0.789	0.831

A value of 1/*n* reflects the adequacy of adsorption for the Freundlich isotherm model (Supong et al. 2019). In this investigation, the 1/*n* values, which are smaller than 1, were found to be 0.202 ± 0.04, 0.207 ± 0.06, 0.214 ± 0.08, and 0.237 ± 0.04 at 298, 308, 318, and 328 K, respectively (Table 1). These findings suggest that materials with a more uniform distribution of surface-active sites are more likely to bind 2,4-D and the Langmuir model is better suited for 2,4-D adsorption onto Fe_3_O_4_@NiS NPs-AC (Supong et al. 2019).

The exterior binding energy distribution is described by the Temkin isotherm model. Their binding forces are greatly influenced by the quantity as well as the distribution of functional groups on Fe_3_O_4_@NiS NPs-AC and 2,4-D (Hasan et al. 2023). Temkin adsorption capabilities at 298, 308, 318, and 328 K were found to be 7.85 ± 1.03, 8.87 ± 1.26, 10.08 ± 1.61, and 6.52 ± 0.95 L/mg, respectively (Table 1). The adsorption heat for physisorption processes is comparatively low, typically less than 8 kJ/mol, as the adsorbate binds to the adsorbent mainly via weak Van der Waals interactions. In case of chemisorption processes, the adsorbate adheres to the surface through the formation of a chemical bond, so they have relatively high adsorption heat (8–16 kJ/mol) (Pandiarajan et al. 2018). In the current study, the values of b (Eq. S5) for 2,4-D adsorption confirmed the physisorption and relatively weak ionic interactions between 2,4-D molecules and Fe_3_O_4_@NiS NPs-AC (Table 1).

Through the D-R isotherm model, it was possible to figure out prominent free energy of the adsorption process and the typical porosity. The adsorption mechanism is thought to be chemical when E (Eq. S8) ranges from 8 to 16 kJ/mol, while it is physical when E is less than 8 kJ/mol (Hasan et al. 2023). It follows that physical adsorption governs 2,4-D adsorption on Fe_3_O_4_@NiS NPs-AC since *E* values were determined as 0.61, 0.67, 0.76, and 0.72 kJ/mol at 298, 308, 318, and 328 K, respectively (Table 1). Based on the reported values of *R*^2^ and the error functions of the equilibrium data for 2,4-D adsorption for all four isotherm models as presented in Table 1, it can be inferred that the Langmuir isotherm model exhibits the most favorable agreement with the experimental data of the adsorption of 2,4-D onto the Fe_3_O_4_@NiS NPs-AC nanocomposite, showing that monolayer coverage is the main mechanism for 2,4-D adsorption.

Table S2 compares the Langmuir adsorption capabilities for 2,4-D removal from aqueous solutions employing various AC based adsorbents. It has been demonstrated that Fe_3_O_4_@NiS NPs-AC possesses a substantially excellent adsorption capability, as indicated by the data in Table S2. Besides, the low-cost and environmentally benign production of a simple, nontoxic, stable, and affordable magnetic nanoadsorbent using a cost-effective and renewable source (*SS* algae) is reported for the first time.

This alga invasion has dangerous impacts on both the economy and the environment. As a result, we suggest an effective way to transform this underutilized algal biomass into an outstanding sorbent for the efficient and fast removal of the hazardous 2,4-D herbicide from aqueous solutions, which contributes significantly to long-term sustainability. We were able to achieve two sustainable goals by removing this harmful organic herbicide by using an inexpensive and environmentally friendly adsorbent prepared from *SS* biomass. Therefore, the synthesis of Fe_3_O_4_@NiS NPs-AC utilizing *SS* biomolecules appears to be a win–win situation, as undesirable collected *SS* is being used for sustainable activity. When comparing this adsorbent with those previously reported, we can see that it can remediate high 2,4-D concentrations (50–200 mg/L) in a reasonable time (3 h) with a high adsorption capacity (208.26 ± 15.75 mg/g). Thus, our adsorbent is economically viable for practical, real-world applications.

### Sorption kinetic study

Adsorption kinetics, as a key element in studying adsorption efficiency, proposes insights into the adsorption mechanism. Batch adsorption studies were performed to examine 2,4-D adsorption kinetics on Fe_3_O_4_@NiS NPs-AC with various initial concentrations (100, 125, and 150 mg/L) at 298 K. The pseudo-first order (Eq. S9), pseudo-second order (Eq. S10), and Elovich (Eq. S11) models were applied to analyze the adsorption kinetics. Additional information about these models and their parameters are provided in Table S3 in supplementary materials.

**Fig. 10:**
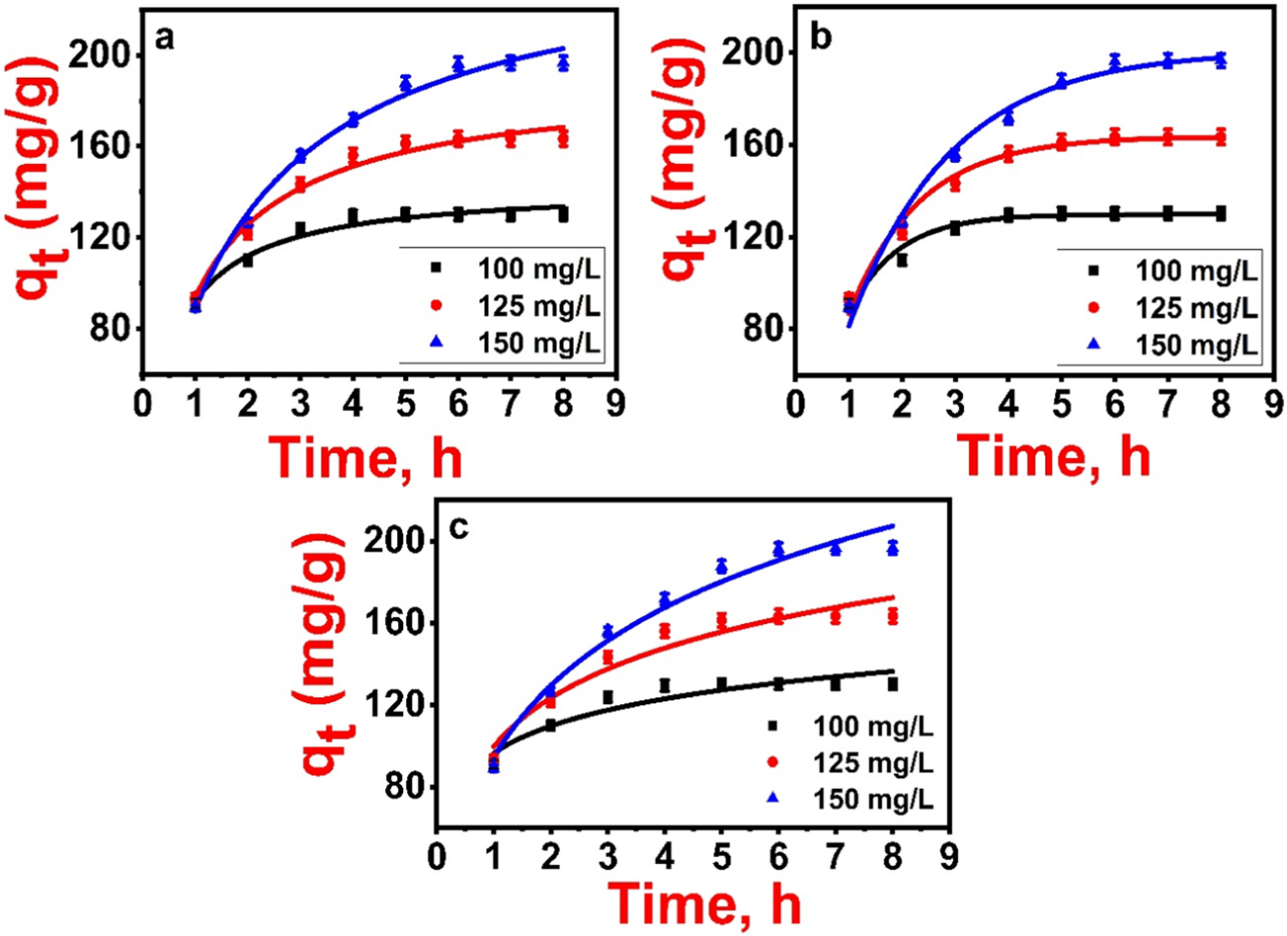
Kinetic models of 2,4-D adsorption on Fe_3_O_4_@NiS NPs-AC: (**a**) pseudo-first order, (**b**) pseudo-second order, and (**c**) Elovich

The nonlinear plots of these kinetic models are displayed in Fig. 10a–c, and Table 2 lists the parameter values for each model. Using the R^2^ and error function values, the fits and correlations of the kinetic models are assessed. For all initial 2,4-D concentrations, the pseudo-second order model (Fig. 10b) shows a slightly higher correlation, according to the *R*^2^ and error function values in Table 2. Additionally, *Δ*q% and MRD% are utilized to validate the kinetic models' fittings at various initial 2,4-D concentrations. Pseudo-second order is confirmed as the better fitting kinetic model since all MRD% and *Δ*q% values are lower. Nevertheless, the almost identical *R*^2^ values for both models also reflect the possibility of chemical and physical adsorption of the 2,4-D adsorption onto Fe_3_O_4_@NiS NPs-AC (Hazrin et al. 2022). In general, the pseudo-second order model exhibits higher *R*^2^ and lower error function values for all initial concentrations, suggesting a greater dominance of chemisorption over physisorption (Hazrin et al. 2022). The pseudo-second-order rate constant decreased with increasing 2,4-D concentration suggesting that the adsorption rate is related to the availability of active sites on the adsorbent surface (Njoku and Hameed 2011). The Elovich kinetic model was utilized as well to study 2,4-D adsorption kinetics (Fig. 10c). Table 2 illustrates the results of computing *α* and *β* which indicated the rapid adsorption characteristics as reflected by the high calculated values of *α*. Considering all initial 2,4-D concentrations, the corresponding values of *α* were also much higher than β, as shown in Table 2. Therefore, it shows how 2,4-D adsorption rate was higher than its desorption rate on Fe_3_O_4_@NiS NPs-AC.

**Table 2:** Kinetic parameters of 2,4-D adsorption onto Fe_3_O_4_@NiS NPs-AC under conditions: 0.075 g adsorbent dose over 100–150 mg/L at pH 5, contact time 1–8 h, and 298 K

Model	Parameter	Initial 2,4-D concentration (mg/L)		
		100	125	150
Pseudo first order	*k*_1_	1.11 ± 0.06	0.75 ± 0.04	0.52 ± 0.03
	*q*_*e*_(cal)	142.75 ± 2.3	189.71 ± 3.87	248.85 ± 6.20
	*R*^2^	0.965	0.979	0.989
	*SSE*	50.90	95.36	116.94
	*χ*^2^	8.48	15.89	19.49
	*MSE*	8.47	15.92	17.14
	adjR^2^	0.959	0.976	0.987
	Δq%	6.784	11.370	18.789
	MRD%	9.58	16.07	26.56
Pseudo second order	*k*_2_	0.013 ± 0.002	0.005 ± 0.0006	0.002 ± 0.0002
	*q*_*e*_(cal)	129.85 ± 1.30	163.73 ± 2.10	201.09 ± 3.12
	*R*^2^	0.966	0.981	0.990
	*SSE*	50.09	85.89	108.10
	*χ*^2^	8.35	14.31	18.02
	*MSE*	8.35	14.29	17.98
	adjR^2^	0.959	0.978	0.988
	Δq%	0.225	0.131	1.624
	MRD%	0.32	0.17	2.27
Elovich	*β*	0.052 ± 0.008	0.028 ± 0.003	0.017 ± 0.002
	*α*	2869.86 ± 2371.23	536.85 ± 199.44	227.71 ± 43.03
	*R*^2^	0.881	0.941	0.974
	*SSE*	173.14	273.13	281.87
	*χ*^2^	28.86	45.52	46.98
	*MSE*	4.93	5.15	6.71
	adjR^2^	0.861	0.931	0.967
Experimental adsorption capacity	*q*_*e*_(exp.)	130.27 ± 0.70	163.45 ± 0.94	196.63 ± 0.65

### Mass transfer models

The study on adsorption kinetics provides insights into the mechanisms involved in mass transfer. The kinetic process of adsorption involves three distinct steps. The initial step is external film diffusion, where the adsorbate moves through the liquid film surrounding the adsorbent. The driving force for external diffusion is the concentration difference between the bulk solution and the adsorbent’s surface. The second step is pore diffusion, which characterizes the movement of the adsorbate within the pores of the adsorbent. The final step is surface diffusion, wherein the adsorbate is captured by the active sites on the adsorbent (Wang and Guo 2020). Hence, it is imperative to investigate whether steps (2) and (3), either individually or in conjunction, serve as the rate-controlling steps in the sorption process of 2,4-D onto Fe_3_O_4_@NiS NPs-AC.

**Fig. 11:**
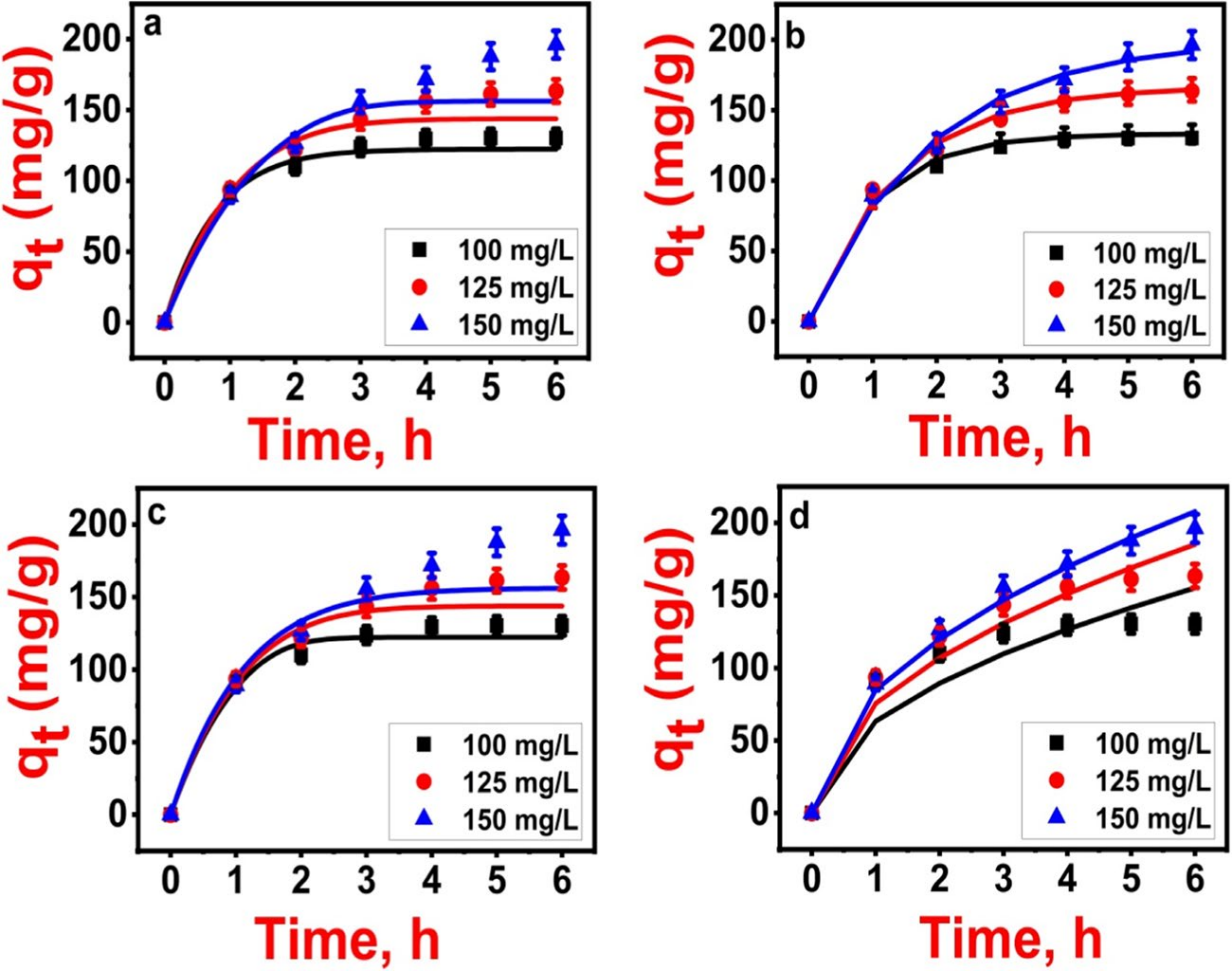
Mass transfer models of 2,4-D adsorption on Fe_3_O_4_@NiS NPs-AC: (**a**) EMS, (**b**) M&W, (**c**) IMS, and (**d**) W&M

In this investigation, four individual resistance mass transfer models were examined. EMT (Eq. S12) and M&W models (Eq. S13), focus on external mass transfer resistance, while IMT (Eq. S14) and W&M models (Eq. S15) are centered on internal mass transfer resistance. A summary of the model equation constants, parameters, and corresponding units is provided in Table S4. To explore EMT and IMS, Langmuir isotherm was employed for the experimental data concerning the kinetics of 2,4-D adsorption. This choice was made as the Langmuir isotherm exhibited the best fit among the equilibrium isotherms considered. Figure 11 displays the nonlinear plots of these models, and their parameters are summarized in Table 3.

**Table 3:** Parameters of mass transfer models for 2,4-D adsorption onto Fe_3_O_4_@NiS NPs-AC

Model	Parameter	Initial 2,4-D concentration (mg/L)		
		100	125	150
EMS	*k*_ext_	1.55 ± 0.07	1.09 ± 0.04	0.77 ± 0.03
	*q*_*e*_ (cal)	122.31 ± 2.50	143.88 ± 3.40	156.29 ± 5.12
	*R*^2^	0.983	0.950	0.882
	*SSE*	216.67	915.36	2893.63
	*χ*^2^	1.79	6.42	18.59
	*MSE*	957.90	17100.79	170883.02
	*Δ*q%	4.520	8.666	14.707
	MRD%	6.11	11.98	20.52
M&W	*k*_M&W_	1.01 ± 0.002	0.715 ± 0.006	0.53 ± 0.002
	*q*_*e*_ (cal)	133.02 ± 3.3	164.38 ± 2.87	191.48 ± 1.20
	*R*^2^	0.994	0.995	0.996
	*SSE*	90.97	112.97	116.40
	*χ*^2^	0.873	1.15	1.02
	*MSE*	169	260.50	267.56
	*Δ*q%	1.498	0.407	1.856
	MRD%	2.11	0.57	2.62
IMS	*k*_int_	0.77 ± 0.005	0.80 ± 0.006	0.84 ± 0.009
	*q*_*e*_ (cal)	122.33 ± 2.37	143.89 ± 1.99	156 ± 4.30
	*R*^2^	0.980	0.950	0.870
	*SSE*	255.80	913.80	3115.14
	*χ*^2^	2.15	6.42	20.22
	*MSE*	1335.17	17040.69	198042.80
	*Δ*q%	4.340	8.472	14.631
	MRD%	6.09	11.97	20.66
W&M	*k*_W&M_	63.34 ± 8.23	75.59 ± 3.56	84.83 ± 1.2
	*q*_*e*_ (cal)	155.16 ± 7.23	185.15 ± 3.24	207.78 ± 1.03
	*R*^2^	0.890	0.951	0.991
	*SSE*	2124.51	1253.48	281.46
	*χ*^2^	23.30	10.58	1.79
	*MSE*	92112.25	32083.97	1616.84
	*Δ*q%	13.610	9.488	4.109
	MRD%	19.11	13.28	5.67
Experimental adsorption capacity	*q*_*e*_ (exp.)	130.27 ± 0.70	163.45 ± 0.94	196.63 ± 0.65

Based on the error analysis and the interpretation of model plots (Fig. 11), it can be concluded that the M&W model provided a superior fit to the experimental kinetic data for 2,4-D adsorption. In addition, the validity of these models was further assessed through the comparison of experimental *q*_*e*_ results with theoretical model calculations. The M&W model has the highest *R*^2^ values and the lowest MRD% and *Δ*q% values among the others. This suggests that, under the specified conditions, the primary limiting step in the adsorption rate is the diffusion of 2,4-D through the boundary liquid layer surrounding Fe_3_O_4_@NiS NPs-AC particles (Wang et al. 2008). The determined *k*_M&W_ values at initial 2,4-D concentrations of 100, 125, and 150 mg/L were computed to be 1.01 ± 0.002, 0.715 ± 0.0006, and 0.53 ± 0.0002 cm/h, respectively. The *k*_M&W_ values exhibited a decline with rising initial 2,4-D concentrations, indicating a slower external mass transfer rate at higher 2,4-D concentrations (Ahmed and Theydan 2013). This implies that the velocity of 2,4-D transport from the liquid phase to the solid phase decreased, while intraparticle diffusion increased with higher initial 2,4-D concentrations, as shown by the increase in the *k*_W&M_ values (Wang et al. 2008) (Table 3). However, these values suggest that the 2,4-D transport from the liquid phase to the solid phase is sufficiently rapid, implying the potential suitability of Fe_3_O_4_@NiS NPs-AC for treating wastewater containing elevated concentrations of 2,4-D.

### Thermodynamics analysis

Employing experimental information along with conventional thermodynamic relationships, fundamental thermodynamic parameters, including Gibbs free energy (*ΔG*°), enthalpy change (*ΔH*°), and entropy change (*ΔS*°), were calculated. The van’t Hoff equation and other equations used for calculating these parameters were listed in Table S5 (Eq. S16-19).

**Fig. 12:**
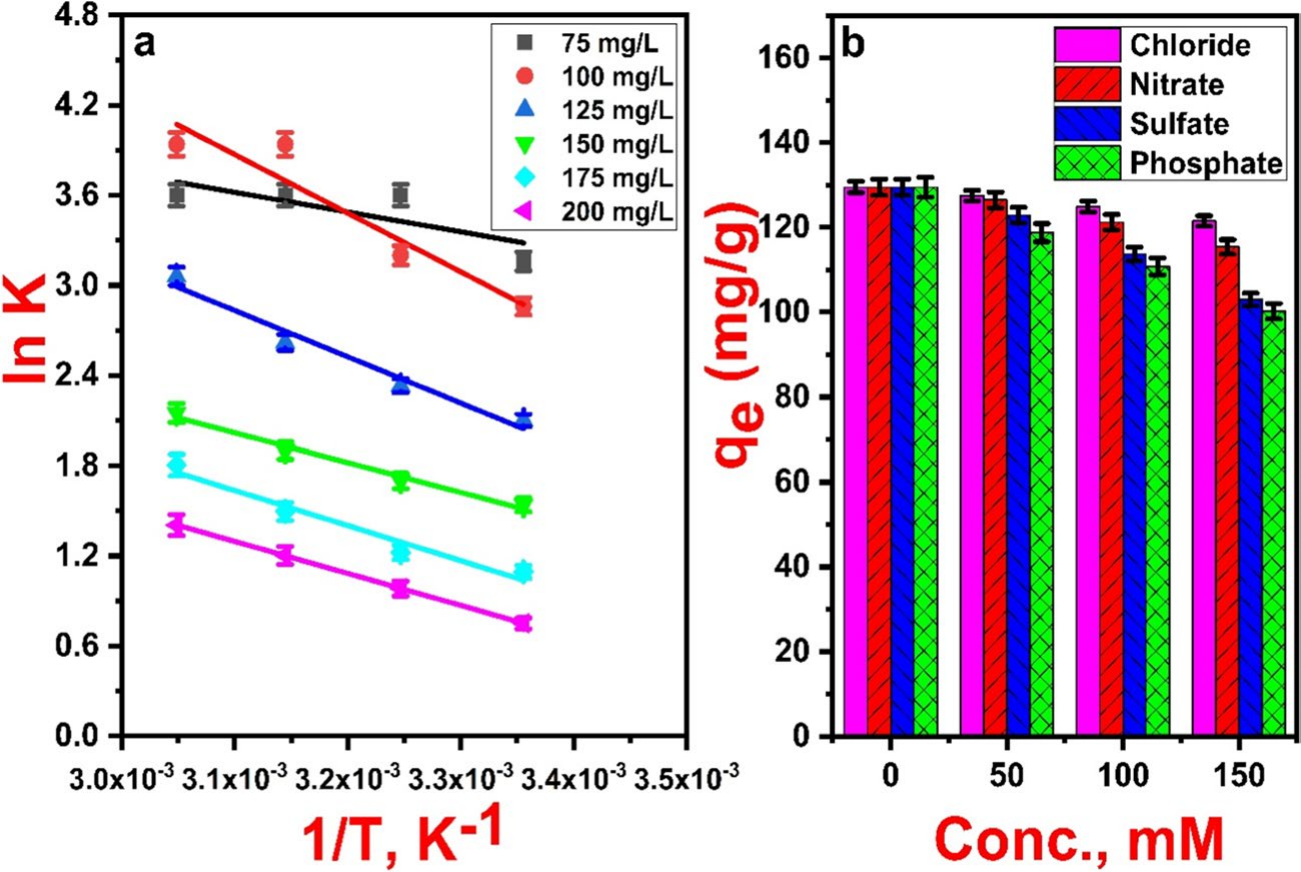
Plot of ln K versus 1/T for thermodynamic analysis effect (**a**) and effect of different coexisting anions on 2,4-D adsorption capacity (**b**)

Figure 12a shows the linear plot of the van’t Hoff equation for different 2,4-D concentrations (75–200 mg/L), and Table 4 summarizes the estimated values for thermodynamic parameters. As observed, the 2,4-D adsorption on Fe_3_O_4_@NiS NPs-AC has been demonstrated to be endothermic and spontaneous by the positive values of *ΔH*° and the negative values of *ΔG*°. At higher temperatures, favorable adsorption can be observed by the moderate decrease of *ΔG*° values. As a result of its porous nature and its accessibility of more surface sites at increased temperatures, the adsorption potential of Fe_3_O_4_@NiS NPs-AC is enhanced. In addition, the estimated values of *ΔH*° highlighted the presence of substantial physical interactions among the solid and bulk phases, particularly in the manner of electrostatic attraction (Salomón et al. 2021). Likewise, the substantial affinities for 2,4-D molecules to Fe_3_O_4_@NiS NPs-AC surface were corroborated by the relatively small value of *ΔS*°; meanwhile, its positive values correlated with greater randomness at the sorbent-sorbate interface (Pandiarajan et al. 2018).

**Table 4:** Thermodynamic parameters for 2,4-D uptake by Fe_3_O_4_@NiS NPs-AC

Parameter	Temp., K	Initial concentration, mg/L					
		75	100	125	150	175	200
*ΔG*° (kJ/mol)	298	− 8.14	− 7	− 5.15	− 3.62	− 2.622	− 1.928
	308	− 8.78	− 8.33	− 6.18	− 4.30	− 3.362	− 2.588
	318	− 9.42	− 9.66	− 7.25	− 4.98	− 4.102	− 3.248
	328	− 10.06	− 10.99	− 8.24	− 5.66	− 4.842	− 3.908
*ΔH*° (kJ/mol)		10.93	32.63	25.54	16.64	19.43	17.74
*ΔS*° (kJ/mol·K)		0.064	0.133	0.103	0.068	0.074	0.066

### Effects of co-existing anions on 2,4-D uptake

Coexisting anions can typically be detected with herbicide effluents that have been discharged into aquatic ecosystems. The removal of a certain pollutant can be restricted by the presence of these ions which cause a higher ionic strength in contaminated water (Mpatani et al. 2021). Thus, it is necessary to research how these coexisting anions impact the selectivity of the adsorbent and the adsorption efficacy of 2,4-D. To demonstrate trends in the selective 2,4 D uptake, four common anions (Cl^−^, NO_3_^−^, SO_4_^2−^, and PO_4_^3−^) were selected at concentrations (50–150 mM) higher than what is found in ordinary water sources (Chaparadza and Hossenlopp 2011). Figure 12b depicts the adsorption capacity of 2,4-D in the presence of Cl^−^, NO_3_^−^, SO_4_^2−^, and PO_4_^3−^ at concentrations between 0 and 150 mM. In the absence of competing anions, an adsorbent dose of 0.75 g/L eliminated 97.12 ± 0.95% of the herbicide from a 100 mg/L solution (Fig. S1). The results in Fig. 12b demonstrate that the co-existence of such anions in the water matrix has a negative effect on the absorption of 2,4-D onto Fe_3_O_4_@NiS NPs-AC. All anions decreased the ability of Fe_3_O_4_@NiS NPs-AC adsorbent to remove 2,4-D. In addition, as the anion concentration increased, the adsorption uptake generally dropped as well (Mpatani et al. 2021). The adsorption removal and capacity of 2,4-D decreased in order Cl^−^ < NO_3_^−^* < *SO_4_^2−^* < *PO_4_^3−^. In the presence of monovalent anions, Cl^−^ and NO_3_^−^, there was a slight effect on the adsorption removal and capacity of 2.4-D. Even under high concentration of both ions, the adsorption capacity decreased by less than 11 ± 1.05% (Fig. S1), and the adsorption capacity decreased from 129.5 ± 1.4 to 115.4 ± 2.3 mg/g (Fig. 12b). Meanwhile, both low and high concentrations of divalent SO_4_^2−^ and trivalent PO_4_^3−^ anions exhibited higher inhibition as the adsorption efficiency was less than 76 ± 1.05% (Fig. S1), and the adsorption capacity dropped from 129.5 ± 1.4 to 100 ± 1.8 mg/g (Fig. 12b). This was because of their larger negative charges, which made them more electrostatically attracted to the surface of Fe_3_O_4_@NiS NPs-AC, hindering 2,4-D from adhering to the surface (Liu et al. 2022). However, even at high concentrations of these competing anions, the adsorption removal and capacity of Fe_3_O_4_@NiS NPs-AC towards 2,4-D remained around 76 ± 1.05% and 100 ± 1.8 mg/g, respectively. Thus, Fe_3_O_4_@NiS NPs-AC has a high adsorption selectivity towards 2,4-D, and the adsorption process is not only based on electrostatic attraction between the adsorbent and 2,4-D. Similar results were described by (Zhang and Han 2022).

### Adsorption of 2,4-D in the real water samples

**Fig. 13:**
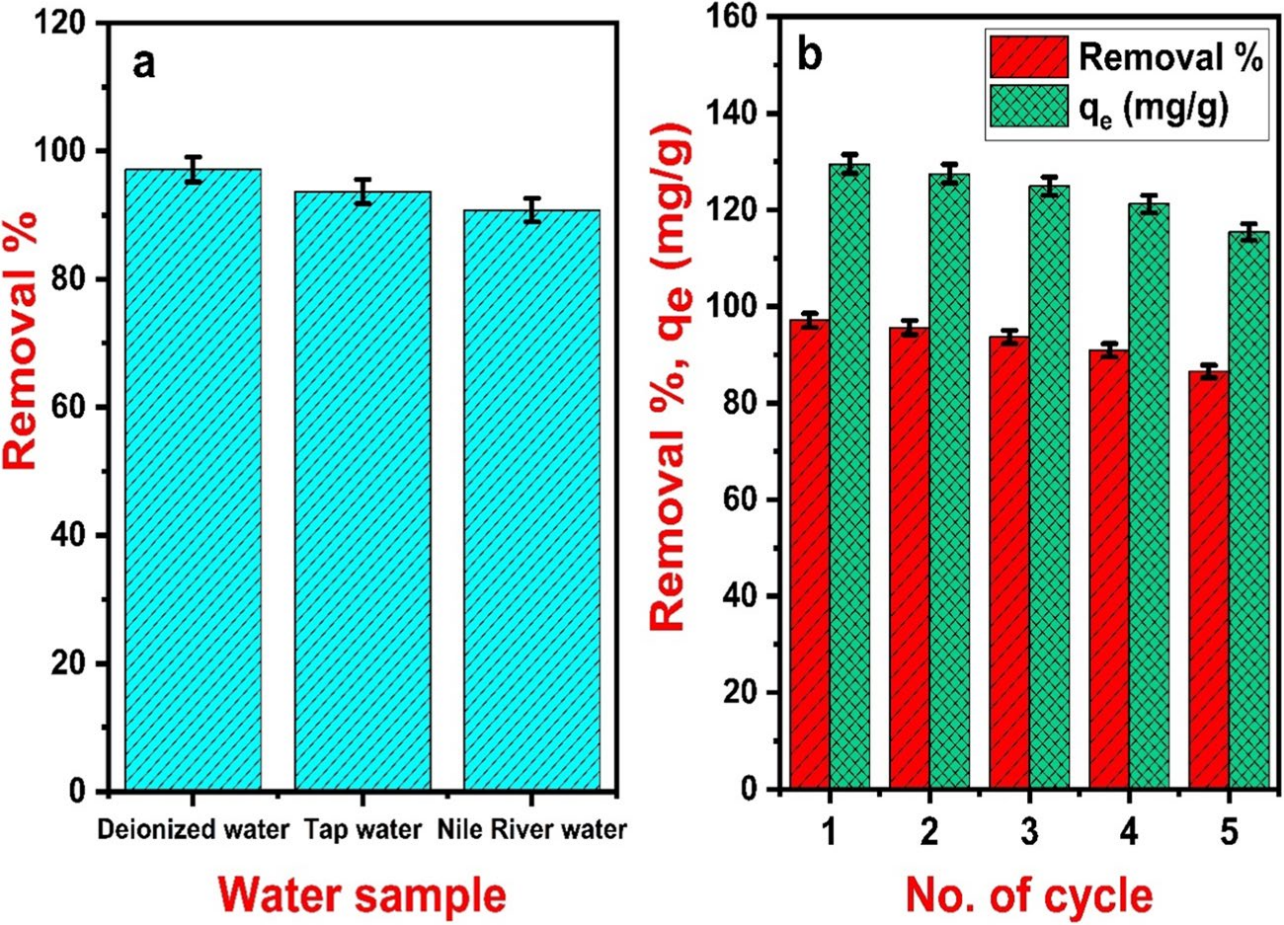
The removal efficiency of 2,4-D in various real water samples (**a**) and removal and adsorption capacity of 2,4-D after five cycles of reuse (**b**)

Aquatic environments exhibit a complex composition, typically comprising suspended solids, natural organic matter, and inorganic salts. Therefore, it is vital for the developed adsorbent to selectively remove the target water pollutant in the broad distribution of these matrix constituents (Tan and Foo 2021). A study of the influence of real water samples on 2,4-D adsorption was conducted to assess the capability of Fe_3_O_4_@NiS NPs-AC for practical applications. 100 mg/L of 2,4-D was introduced to both tap water and Nile River water samples, and the pH values were adjusted to 5 by adding 0.1 M HCl after adding the adsorbent. Figure 13a displays the removal percentages of 2,4-D from deionized water, tap water, and Nile River water using Fe_3_O_4_@NiS NPs-AC. It can be observed that the adsorptive uptakes of 2,4-D by Fe_3_O_4_@NiS NPs-AC were decreased in both tap water and Nile River water. Tap water exhibited a removal decline of about 4 ± 1.87%, whereas Nile River water showed a 7 ± 1.82% removal drop. This removal decline can be assigned to the competition between 2,4-D molecules and water matrix components for the adsorption sites on the adsorbent surface (Tan and Foo 2021). Similar trends were reported by (Tan and Foo 2021; Lazarotto et al. 2021; Zhang and Han 2022). Considering these results, Fe_3_O_4_@NiS NPs-AC is a viable adsorbent for 2,4-D adsorption from real water effluents.

### Reusability and stability of Fe_3_O_4_@NiS NPs-AC adsorbent

Reusable adsorbents are essential for effective water treatment. Evaluation of adsorbent cycle performance in practical settings is essential for assessing long-term viability. Fe_3_O_4_@NiS NPs-AC was regenerated five times in a reusability study using a 2,4-D concentration of 100 mg/L to assess its potential for repeated use. According to Fig. 13b, as the cycle number rises, the adsorption removal and capacity of 2,4-D slightly decline. After five cycles of regeneration, the adsorbent's adsorption removal decreases by 11 ± 1.5% compared to the virgin material. Thus, Fe_3_O_4_@NiS NPs-AC is an economically viable adsorbent for water treatment since it can be recycled multiple times without losing its capacity.

Based on AAS data, the amount of leached iron from the solution ranged from 0.12 ± 0.03 to 0.28 ± 0.06 mg/L after five cycles of regeneration (Table S8). These results support the high stability of Fe_3_O_4_@NiS NPs-AC in multi-cycle use and demonstrate the outstanding stability and broad potential applications of Fe_3_O_4_@NiS NPs-AC. Thus, it is useful for the remediation of 2,4-D from wastewater, along with lowering pollution levels and promoting sustainability.

### The adsorption mechanism of 2,4-D onto Fe_3_O_4_@NiS NPs-AC

**Fig. 14:**
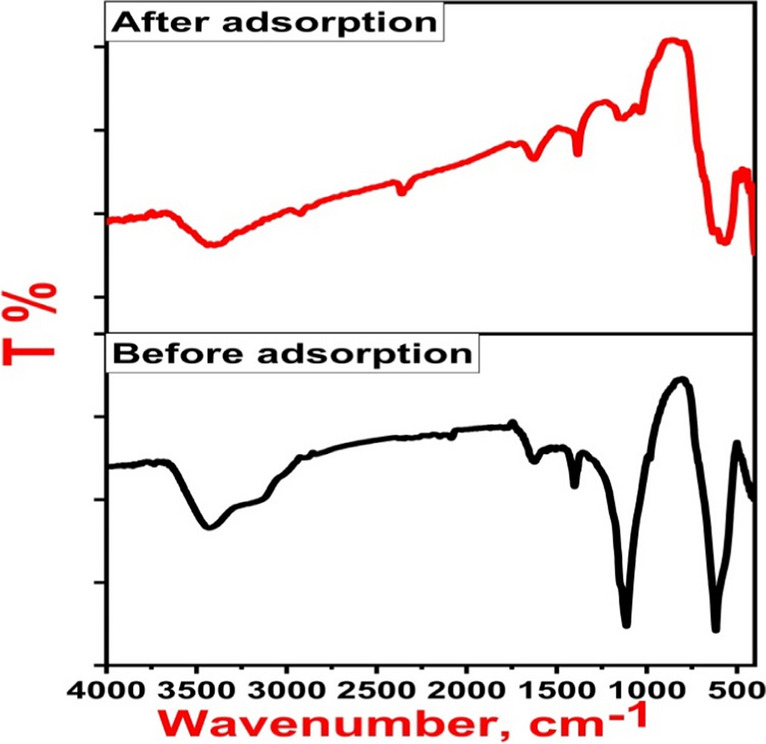
FTIR of Fe_3_O_4_@NiS NPs-AC before and after 2,4-D adsorption

**Fig. 15:**
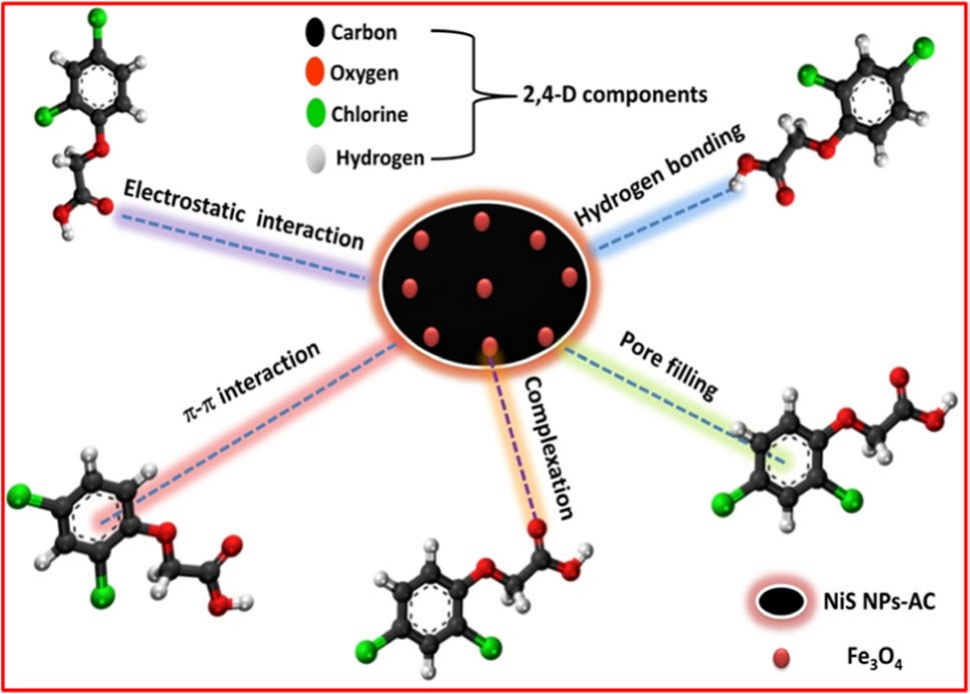
Mechanism of 2,4-D adsorption onto Fe_3_O_4_@NiS NPs-AC

Incorporating the different characterization results of the prepared magnetic nanoadsorbent with the conclusions drawn from pH, equilibrium, kinetic, and thermodynamic studies of 2,4-D adsorptive behavior on Fe_3_O_4_@NiS NPs-AC surface, it was obvious that adsorption mechanisms included complex physicochemical variations (Pandiarajan et al. 2018). A multi-mechanism manner may be proposed for the 2,4-D adsorption onto Fe_3_O_4_@NiS NPs-AC depending on pH dependency, surface area and pore size measurements, FTIR, and adsorption theory studies. Electrostatic interactions, pore-filling, hydrogen bonding, π-π interactions, and complexation comprise the mechanisms that controlled 2,4-D adsorption onto Fe_3_O_4_@NiS NPs-AC (Tan et al. 2015). 2,4-D molecules mainly existed in neutral form with a considerable amount of deprotonation at an acidic pH (Rambabu et al. 2021). Additionally, in this low pH environment, Fe_3_O_4_@NiS NPs-AC had a positive surface charge. This caused the 2,4-D molecules and the Fe_3_O_4_@NiS NPs-AC surface to engage electrostatically. The positively charged active centers of Fe_3_O_4_@NiS NPs-AC surface primarily electrostatically attracted the herbicide's negatively polarized C = O group (Pandiarajan et al. 2018). On the other hand, in an alkaline environment, the deprotonation of 2,4-D molecules increased, and the Fe_3_O_4_@NiS NPs-AC had a negative charge, leading to a strong repulsion between them, explaining the drop in adsorption removal. Based on the surface area and pore size measurements, Fe_3_O_4_@NiS NPs-AC exhibited a significant abundance of mesoporous structure. In addition, the molecular size of 2,4-D was theoretically determined as 1.54 × 0.56 × 0.22 nm (Binh and Nguyen 2020). As a result, the 2,4-D molecular size and the pore size of Fe_3_O_4_@NiS NPs-AC are comparable, meaning that the 2,4-D can engage in the mesopores of Fe_3_O_4_@NiS NPs-AC. Besides, the respective specific surface area and total pore volume of Fe_3_O_4_@NiS NPs-AC were reduced from 288.439 m^2^/g and 0.192 cm^3^/g before adsorption to 210.50 m^2^/g and 0.068 cm^3^/g after 2,4-D adsorption. This reduction demonstrated the diffusion of 2,4-D molecules into the interior part of Fe_3_O_4_@NiS NPs-AC through pore-filling effect (Binh and Nguyen 2020; Mpatani et al. 2021). To support the adsorption mechanism of 2,4-D, FTIR analysis was conducted, in which alterations in the FTIR spectra of Fe_3_O_4_@NiS NPs-AC before and after 2,4-D adsorption were observed (Fig. 14). According to the FTIR data, the functional groups were obviously involved in 2,4-D adsorption. In particular, the stretching and bending vibrations of hydroxyl groups were shifted from 3431 and 1397 cm^−1^ to 3412 and 1387 cm^−1^, respectively. This red shift demonstrates the formation of hydrogen bonds between hydrogen atoms of the hydroxyl groups of the adsorbent with O atom from carboxylated and carbonyl groups of 2,4-D molecules (Liu et al. 2022; Sayğılı and Sayğılı 2022; Binh and Nguyen 2020; Zhang and Han 2022). In addition, the obvious red shift and reduction of the FTIR bands related to NiS NPs and Fe_3_O_4_ components after 2,4-D adsorption indicated complexation or electrostatic attraction phenomena during the adsorption process (Demiti et al. 2022; Sayğılı and Sayğılı, 2022; Zhang and Han 2022). Moreover, the appearance of new weak bands at 2929.4, 1748, and 1043 cm^−1^ which are assigned to the stretching vibrations of C−H, C = O, and C−O−C of 2,4-D molecule, respectively, is strong evidence for the successful capture of 2,4-D by Fe_3_O_4_@NiS NPs-AC (Zhang and Han 2022; Chidambaram 2016; Kırbıyık et al. 2017). The findings validated the contribution of chemisorption and aligned with the kinetic data (Chidambaram 2016). Apart from this, the herbicide was effectively sequestered onto surface Fe_3_O_4_@NiS NPs-AC by π-π interactions that occurred between the aromatic moiety of 2,4-D and the graphitic adsorbent surface (Rambabu et al. 2021). The adsorption isotherm, kinetics, and thermodynamic studies also revealed that the adsorption process was carried out by both physical and chemical adsorption. Thus, it was suggested that Fe_3_O_4_@NiS NPs-AC mostly wrapped 2,4-D through electrostatic attraction, pore-filling, hydrogen bonding, π-π interactions, and complexation as shown in Fig. 15.

### Cost estimation of Fe_3_O_4_@NiS NPs-AC

Additional information about the adsorbent’s applicability and economic feasibility for real-world applications can be obtained via cost assessment. To evaluate the commercial advantages of Fe_3_O_4_@NiS NPs-AC nanocomposite, its production cost was computed. Transportation of algae, chemicals (H_2_SO_4_, 98%, NaHCO_3_, Na_2_S, NiCl_2_·2H_2_O, C_2_H_5_OH, Fe(NO_3_)_3_·9H_2_O, FeSO_4_·6H_2_O, and NH_3_, 33%), and power needed in the Fe_3_O_4_@NiS NPs-AC production process were considered during the calculation of the production costs. The SS alga is readily available for free because it is a natural product that is collected from the seashore. However, transportation, electricity, and water handling expenses would be factored in. Furthermore, the yield of every stage in the synthesis process was considered when determining the market prices of the chemical reagents utilized for synthesizing the Fe_3_O_4_@NiS NPs-AC nanocomposite. Table 5 displays the predicted cost of synthesizing Fe_3_O_4_@NiS NPs-AC nanocomposite utilizing *SS* alga. The production cost was estimated to be around 1.615 USD/kg of Fe_3_O_4_@NiS NPs-AC, which is much lower than the market price of AC (up to 5.76 USD/kg of AC) as well as other types of adsorbents, as shown in Table S2. This suggests that Fe_3_O_4_@NiS NPs-AC nanocomposite is commercially feasible. According to the findings of this investigation, high adsorption proficiency in 2,4-D removal is feasible by utilizing our adsorbent, which is an eco-friendly, sustainable, and cheap adsorbent.

**Table 5:** Estimated production cost (USD/kg) of Fe_3_O_4_@NiS NPs-AC nanocomposite

Component	Estimated price (USD/kg)
Transportation	0.015
Chemicals	1.05
Electrical consumption (charge rate of electricity in Egypt = 0.016 UDS/KWh)	0.55
Total estimated cost	1.615

## Conclusion

In this study, we proposed and developed a potential method for the fabrication of magnetic nanoadsorbent which exhibited superior adsorption performance for 2,4-D. The substantially widespread and cost-effective *SS* biomass was utilized to develop AC. The prepared AC was successfully incorporated with NiS NPs and then magnetized through co-precipitation approach. Various characterization methods, involving FTIR, XRD, SEM, and EDX, have been employed to demonstrate the uniform distribution as well as the successful development of nanoparticles on the surface of AC. The improved adsorption capacity of Fe_3_O_4_@NiS NPs-AC for 2,4-D was demonstrated by comparing with the adsorption performances of AC, NiS NPs, and NiS NPs-AC. In accordance with the equilibrium results, 2,4-D followed the Langmuir isotherm model with a maximum adsorption capacity of 208.26 ± 15.75 mg/g, which is relatively high in comparison with the published data. The pseudo-second-order model more accurately describes 2,4-D adsorption kinetics, and the thermodynamic results indicated that the adsorption process was spontaneous, endothermic, and more favorable at high temperatures. Adsorption percentage was determined as higher than 89 ± 0.94% after the reusability tests of Fe_3_O_4_@NiS NPs-AC for 5 cycles. In the end, it is assigned through the adsorption studies that hydrogen bonds, electrostatic interactions, and π-π stacking between the adsorbent and 2,4-D are the primary contributors to adsorption. Considering its facile synthesis method, excellent adsorption capacity, and the reusability potential, Fe_3_O_4_@NiS NPs-AC could serve as a highly economical adsorbent for the successful remediation of 2,4-D from wastewater.

### Supplementary Information

Below is the link to the electronic supplementary material.

Supplementary file1 (DOC 16083 KB)

## Data Availability

Data details are available for this work upon request to the authors.
