# Supplementary material for: Sustainable synthesis of magnetic Sargassum siliquastrum activated carbon loaded with NiS nanorods for adsorption of 2,4-D herbicide
Source: Environ Sci Pollut Res Int. 2024 Jan 20;31(9):13246–69. doi: 10.1007/s11356-024-31987-x (PMC10881655; doi:10.1007/s11356-024-31987-x)
Supplement: Supplementary file 1 — Supplementary file1 (DOC 16083 KB) [file 11356_2024_31987_MOESM1_ESM.doc]

**Sustainable synthesis of magnetic *Sargassum siliquastrum* activated carbon loaded with NiS nanorods for adsorption of 2,4-D herbicide**

Ibrahem M. A. Hasan, Fawzy H. Assaf, Ahmed R. Tawfik*

Chemistry Department, Faculty of Science, South Valley University, Qena 83523, Egypt

*Corresponding authors: Tel +201025795556, E-mail: [atawfik2018@gmail.com](mailto:atawfik2018@gmail.com) (Ahmed R. Tawfik).

**Supplementary material**

**Table S 1** Non-linear forms of isotherms used for fitting the adsorption data.

| **Model** | **Nonlinear form** | **Equation No.** | **Ref.** |
| --- | --- | --- | --- |
| Langmuir isotherm | q_e_ = (q_m_*K_L_*C_e_/(1 + K_L_*C_e_))  R_L_ = 1/(1 + K_L_*C_ᴏ_) | Eq. S 1  Eq. S 2 |  |
| Freundlich isotherm | q_e_ = K_F_*C_e_^1/n^ | Eq. S 3 |  |
| Temkin isotherm | q_e_ = B*ln(A*C_e_)  B = RT/b | Eq. S 4  Eq. S 5 | (Wang and Guo, 2020a) |
| D-R isotherm | q_e_ = q_s_*exp(-K* ε^2^)  ε = RT*ln(1 + 1/C_e_)  E = (1/√2K) | Eq. S 6  Eq. S 7  Eq. S 8 |  |

Where q_e_ is experimental adsorption capacity (mg/g) at equilibrium; q_m_ is the maximum adsorption capacity (mg/g); K_L_ is Langmuir constant (L/g); C_e_ is equilibrium concentration (mg/L); C_o_ is the initial concentration (mg/L); K_F_ is Freundlich model constant; 1/n is heterogeneity factor constant; R is universal gas constant; T is temperature (K); A is equilibrium binding constant (L/mg); b is Temkin constant associated with enthalpy of adsorption; K is a constant associated with adsorption energy; q_s_ is the adsorption capability (mg/g); ε is a representation of the Polanyi potential; E (kJ/mol.) is defined as the free energy change required to transfer 1 mole of material from solution to solid surface.

**Table S 2** Comparison between Fe_3_O_4_@NiS NPs-AC and other AC based adsorbents used to remove 2,4-D.

| **Adsorbent** | **SSA^a^ (m^2^/g)** | **Operating conditions** | **q_max_ (mg/g)** | **Kinetic model** | **Mechanism** | **Cost ($/kg)** | **Ref.** |
| --- | --- | --- | --- | --- | --- | --- | --- |
| **GAC^b^** | 731.48 | T = 303 K, pH = 3–3.8, conc. = 50–300 mg/L, time = 8 h, dose = 1 g/L | 181.82 | Pseudo-second order, | -------------- | ---------- | (Salman and Hameed, 2010) |
| **ACF-Fe_3_O_4_^c^** | ------- | T = 288 K, pH = 7, conc. = 15–100 mg/L, time = 6 h, dose = 0.33 g/L | 51.10 | Pseudo-second order | π-π bonds, H-bonds and electrostatic forces | ---------- | (Demiti et al., 2022) |
| **OPAC^d^** | 1779.48 | T = 298 K, pH = 6.22, conc. = 50–300 mg/L, time = 24 h, dose = 3 g/L | 71.94 | Pseudo-second order | -------------- | ---------- | (Angın and Güneş, 2021) |
| **LEFBAC^e^** | 1065.65 | T = 303 K, pH = 2, conc. = 50–400 mg/L, time = 24 h, dose = 2 g/L | 261.2 | Pseudo-second order | -------------- | ---------- | (Njoku and Hameed, 2011) |
| **GCN-P^f^** | 182.4 | T = 303 K, pH = 2, conc. = 9–300 mg/L, time = 24 h, dose = 0.4 g/L | 77 | Multi-exponential | -------------- | ---------- | (Khoshnood and Azizian, 2012) |
| **GCN-C^g^** | 27.4 | T = 303 K, pH = 2, conc. = 9–300 mg/L, time = 24 h, dose = 0.4 g/L | 33 | Multi-exponential | -------------- | ---------- | (Khoshnood and Azizian, 2012) |
| **Fe@PWHC^h^** | ----------- | T = 333 K, pH = 2, conc. = 10–500 mg/L, time = 24 h, dose = 0.2 g/L | 101.1 | ------------- | π-π bonds, H-bonds and electrostatic forces | ---------- | (Sayğılı and Sayğılı, 2022) |
| **CCB^i^** | 298.01 | T = 298 K, pH = 2, conc. = 20–60 mg/L, time = 15 h, dose = 1.5 g/L | 37.4 | Pseudo-second order | pore-filling π- π EDA bonds and H-bonds | 56 | (Binh and Nguyen 2020) |
| **PSHAC^j^** | 737.90 | T = 303 K, pH = 2, conc. = 50–400 mg/L, time = 24 h, dose = 1 g/L | 260.79 | Pseudo-second order | -------------- | ---------- | (Njoku et al., 2013) |
| **DPC-AC^k^** | 947 | T = 303 K, pH = 2, conc. = 50–250 mg/L, time = 1.5 h, dose = 0.15 g/L | 50.25 | Pseudo-second order | π- π bonds, H-bonds, electrostatic forces, weak van der Waals forces | 3 | (Rambabu et al., 2021) |
| **ABRAC^l^** | 798.752 | T = 298 K, pH = 4, conc. = 25–100 mg/L, time = 4 h, dose = 0.4 g/L | 241.7 | Linear driving force | Electrostatic forces, π-π bonds, H-bonds, n-π interactions | 2.39-5.95 | (Lazarotto et al., 2021) |
| **Fe_3_O_4_@NiS NPs-AC** | **288.439** | **T = 328 K, pH = 5, conc. = 50–150 mg/L, time = 3 h, dose = 0.75 g/L** | **208.26±15.75** | **Pseudo-second order** | **Electrostatic forces, pore-filling, H-bonding, π-π bonds, complexation** | **1.615** | **This study** |

^a^Specific surface area, ^b^Granular activated carbon, ^c^Activated carbon fiber functionalized with iron oxide magnetic nanoparticles, ^d^Orange pulp activated carbon, ^e^Langsat empty fruit bunch activated carbon, ^f^Graphitic carbon nanostructures from filter paper, ^g^Graphitic carbon nanostructures from cotton, ^h^Magnetic pomegranate waste hydrochar, ^i^Corn cob biochar, ^j^Pumpkin seed hull activated carbon, ^k^Date palm coir activated carbon, ^l^Agaricus bisporus residue activated carbon.

**Table S 3** Non-linear forms of kinetic models used for fitting the adsorption data.

| **Model** | **Nonlinear form** | **Equation No.** | **Ref.** |
| --- | --- | --- | --- |
| **Pseudo-first order** | q_t_ = q_e_*(1 - exp(-k_1_*t)) | Eq. S 9 |  |
| **Pseudo-second order** | q_t_ = (q_e_*k_2_*t/(1 + q_e_*K_2_*t)) | Eq. S 10 | (Wang and Guo, 2020b) |
| **Elovich model** | q_t_ = (1/β)*ln(1 + α*β*t) | Eq. S 11 |  |

Where q_e_ is equilibrium uptake capacity (mg/g); q_t_ is uptake capacity at time t (mg/g); k_1_ is pseudo-first-order rate constant (1/h); k_2_ is pseudo-second-order rate constant (g/(mg.h)); α is initial adsorption rate (mg/(g.h)); β is the desorption coefficient related to the extent of surface coverage and activation energy for chemisorption (g/mg).

**Table S 4** Mass transfer models used for fitting the adsorption kinetic data.

| **Model** | **Nonlinear form** | **Equation No.** | **Ref.** |
| --- | --- | --- | --- |
| **EMT** | (dq_t_/dt) = k_ext_*(C_t_ - C_et_) | Eq. S 12 |  |
| **M&W** | q_t_ = (C_ο_/m_s_)*(1 - exp(-k_M&W_*S*t)) | Eq. S 13 | (Wang and Guo, 2020b) |
| **IMT** | (dq_t_/dt) = k_int_*(q_et_ - q_t_) | Eq. S 14 |  |
| **W&M** | q_t_ = k_W&M_*****t^1/2^ | Eq. S 15 |  |

Where q_t_ is uptake capacity (mg/g) at time t; k_ext_ is universal external mass transfer coefficient (L/(g.h)); C_t_ is adsorbate concentration at time t (mg/L); C_et_ is equilibrium adsorbate concentration at the adsorbent surface (mg/L); C_0_ is initial adsorbate concentration (mg/L); m is adsorbent mass (g); m_s_ is mass of adsorbent per unit volume of solution (g/L); k_M&W_ is mass transfer coefficient (cm/h); S: outer surface of adsorbent per unit volume (1/cm); q_et_ is equilibrium adsorption capacity in the pores of the adsorbent (mg/g); k_int_ is internal mass transfer rate constant (1/h); k_W&M_ is intraparticle diffusion coefficient (mg/(g.h^1/2^)).

**Table S 5** Equations used for estimation of thermodynamic parameters.

| **Thermodynamic parameter** | **Equation** | **Equation No.** | **Ref.** |
| --- | --- | --- | --- |
| **Equilibrium constant** | K = (1000*K_L_*MW*[ads.]°/γ) | Eq. S 16 |  |
| **Gibbs free energy** | ΔG° = -RT*ln K | Eq. S 17 |  |
|  | ΔG^o^ = ΔH^o^ - TΔS^o^ | Eq. S 18 | (Lima et al., 2019) |
| **Van’t Hoff plot** | ln K = ΔS°/R - ΔH°/RT | Eq. S 19 |  |

Where K is the adsorption equilibrium constant (dimensionless); K_L_ is Langmuir constant (L/mg); MW is the molecular weight of adsorbate; [ads.]° is the standard concentration of the adsorbate (1 mol./L); γ is the coefficient of activity (dimensionless); R is the ideal gas constant [8.314 J/(mol⋅K)]; T is the absolute temperature (K); ΔG^o^ (kJ/mol.), ΔS^o^ (J/(mol.K)), and ΔH^o^ (kJ/mol.) are the change in Gibbs free energy, entropy, and enthalpy, respectively.

**Table S 6** Equations used for estimation of model validation.

| **Equation** | **Equation No.** | **Ref.** |
| --- | --- | --- |
| **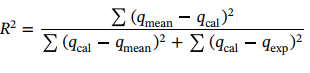** | Eq. S 20 | (Wang and Guo, 2020b) |
| **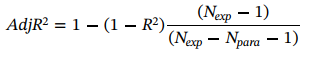** | Eq. S 21 |
| **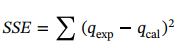** | Eq. S 22 |
| **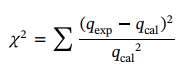** | Eq. S 23 |
| **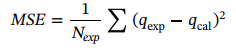** | Eq. S 24 |
| 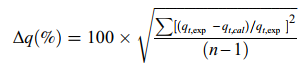 | Eq. S 25 |  |
| MRD (%) = 100/N × ∑q_exp_ – q_cal_/q_exp_ | Eq. S 26 | (Hashisho et al., 2008) |

**Table S 7** Physico-chemical properties of tap water and Nile River water samples.

| **Sample** | **Tap water** | **Nile river water** |
| --- | --- | --- |
| **Ph** | 7.2 | 7.87 |
| **TDS (mg/L)** | 184.5 | 239 |
| **Total hardness as mg CaCO_3_/L** | 70 | 166 |
| **Conductivity (µS)** | 304 | 447 |

**Table S 8 The amount of leached iron in five regeneration cycles.**

| **Cycle** | **1** | **2** | **3** | **4** | **5** |
| --- | --- | --- | --- | --- | --- |
| **Leached iron (mg/L)** | 0.12±0.03 | 0.16±0.04 | 0.23±0.06 | 0.28±0.08 | 0.25±0.04 |





**Fig. S 1** Effect of different coexisting anions on the adsorption removal of 2,4-D.

**References**

Angın, D., Güneş, S. (2021). The usage of orange pulp activated carbon in the adsorption of 2, 4-dichlorophenoxy acetic acid from aqueous solutions. *International Journal of Phytoremediation*, *23*(4), 436-444.

Binh, Q. A., Nguyen, H. H. (2020). Investigation the isotherm and kinetics of adsorption mechanism of herbicide 2, 4-dichlorophenoxyacetic acid (2, 4-D) on corn cob biochar. *Bioresource Technology Reports*, *11*, 100520.

Demiti, G. M. M., Barbosa de Andrade, M., Marcuzzo, J. S., Vieira, M. F., Bergamasco, R. (2022). A novel magnetic adsorbent from activated carbon fiber and iron oxide nanoparticles for 2, 4-D removal from aqueous medium. *Environmental Technology*, 1-19.

Hashisho, Z., Emamipour, H., Rood, M. J., Hay, K. J., Kim, B. J., Thurston, D. (2008). Concomitant adsorption and desorption of organic vapor in dry and humid air streams using microwave and direct electrothermal swing adsorption. *Environmental science & technology*, *42*(24), 9317-9322.

Khoshnood, M., Azizian, S. (2012). Adsorption of 2, 4-dichlorophenoxyacetic acid pesticide by graphitic carbon nanostructures prepared from biomasses. *Journal of Industrial and Engineering Chemistry*, *18*(5), 1796-1800.

Lazarotto, J. S., da Boit Martinello, K., Georgin, J., Franco, D. S., Netto, M. S., Piccilli, D. G., Dotto, G. L. (2021). Preparation of activated carbon from the residues of the mushroom (Agaricus bisporus) production chain for the adsorption of the 2, 4-dichlorophenoxyacetic herbicide. *Journal of Environmental Chemical Engineering*, *9*(6), 106843.

Lima, E. C., Hosseini-Bandegharaei, A., Moreno-Piraján, J. C., Anastopoulos, I. (2019). A critical review of the estimation of the thermodynamic parameters on adsorption equilibria. Wrong use of equilibrium constant in the Van't Hoof equation for calculation of thermodynamic parameters of adsorption. *Journal of molecular liquids*, *273*, 425-434.‏

Njoku, V. O., Foo, K. Y., Hameed, B. H. (2013). Microwave-assisted preparation of pumpkin seed hull activated carbon and its application for the adsorptive removal of 2, 4-dichlorophenoxyacetic acid. *Chemical Engineering Journal*, *215*, 383-388.

Njoku, V. O., Hameed, B. H. (2011). Preparation and characterization of activated carbon from corncob by chemical activation with H_3_PO_4_ for 2, 4-dichlorophenoxyacetic acid adsorption. *Chemical Engineering Journal*, *173*(2), 391-399.

Rambabu, K., AlYammahi, J., Bharath, G., Thanigaivelan, A., Sivarajasekar, N., Banat, F. (2021). Nano-activated carbon derived from date palm coir waste for efficient sequestration of noxious 2, 4-dichlorophenoxyacetic acid herbicide. *Chemosphere*, *282*, 131103.

Salman, J. M., Hameed, B. H. (2010). Adsorption of 2, 4-dichlorophenoxyacetic acid and carbofuran pesticides onto granular activated carbon. *Desalination*, *256*(1-3), 129-135.

Sayğılı, G. A., Sayğılı, H. (2022). Fabrication of a magnetic hydrochar composite via an in situ one-pot hydrocarbonization strategy for efficient herbicide removal. *Diamond and Related Materials*, *128*, 109302.

Wang, J., Guo, X. (2020a). Adsorption isotherm models: Classification, physical meaning, application and solving method. Chemosphere, 258, 127279.‏

Wang, J., Guo, X. (2020b). Adsorption kinetic models: Physical meanings, applications, and solving methods. *Journal of Hazardous materials*, *390*, 122156.
